# Sodium hexafluorophosphate mediated enhancement of electrical and electrochemical properties of poly(vinyl alcohol)–chitosan solid polymer electrolytes for EDLCs†

**DOI:** 10.1039/d5ra02897c

**Published:** 2025-07-11

**Authors:** Vipin Cyriac, Ismayil, Kuldeep Mishra, Ankitha Rao, Riyadh Abdekadir Khellouf, Saraswati P. Masti, I. M. Noor

**Affiliations:** a Department of Physics, Manipal Institute of Technology, Manipal Academy of Higher Education Manipal 576104 Karnataka India ismayil.mit@manipal.edu ismayil.486@gmail.com +91 98454 97546; b Symbiosis Institute of Technology (SIT), Symbiosis International (Deemed University) (SIU) Pune 412115 Maharashtra India; c Department of Electronics and Communication, Manipal Institute of Technology, Manipal Academy of Higher Education Manipal 576104 Karnataka India; d Centre of Polymer Systems, University Institute, Tomas Bata University in Zlin Tr. T. Bati 5678 760 01 Zlin Czech Republic; e Department of Chemistry, Karnataka University's Karnataka Science College Dharwad Karnataka 580001 India; f Ionic Materials and Energy Devices Laboratory, Physics Department, Faculty of Science, Universiti Putra Malaysia 43400 UPM Serdang Selangor Darul Ehsan Malaysia; g Physics Division, Centre for Foundation Studies in Science of Universiti Putra Malaysia, Universiti Putra Malaysia 43400 Serdang Selangor Darul Ehsan Malaysia

## Abstract

A free-standing, flexible and biodegradable biopolymer electrolyte (BPE) derived from a poly(vinyl alcohol) (PVA)–chitosan (CS) blend immobilizing sodium hexafluorophosphate (NaPF_6_) salt was fabricated *via* solution casting method. The effect of salt concentration on the structural, electrical, and electrochemical properties of the electrolyte was systematically investigated. X-ray diffraction (XRD) and Fourier-transform infrared (FTIR) spectroscopy were used to ascertain the microstructural changes in the polymer matrix including the complexation of PVA, CS, and NaPF_6_. Electrochemical impedance spectroscopy (EIS) measurements revealed that the BPE containing 40 wt% NaPF_6_ exhibited the highest conductivity (6.94 ± 0.04) × 10^−5^ S cm^−1^, which was three-order enhancement over the pristine system. The ion transport behaviour, interpreted through the Schütt and Gerdes (S–G) model, revealed that the ionic conductivity of the SPE system is strongly influenced by both the concentration of charge carriers and their mobility. The electrolyte displayed a predominant ionic nature with an electrochemical stability window of ∼3.25 V. When incorporated into an Na-ion EDLC, the optimized electrolyte sample provided a specific capacitance of 42.65 F g^−1^, energy density of 5.4 W h kg^−1^, and power density of 95 W kg^−1^, as determined by galvanostatic charge–discharge (GCD) tests performed at 0.05 mA g^−1^.

## Abstract 2

A NaPF_6_-complexed PVA–chitosan solid polymer electrolyte was developed. Ion dynamics were studied using multiple formalisms. The EDLC device delivered 42.65 F g^−1^ specific capacitance, 5.4 W h kg^−1^ energy density, and 95 W kg^−1^ power density.

## Graphical abstract

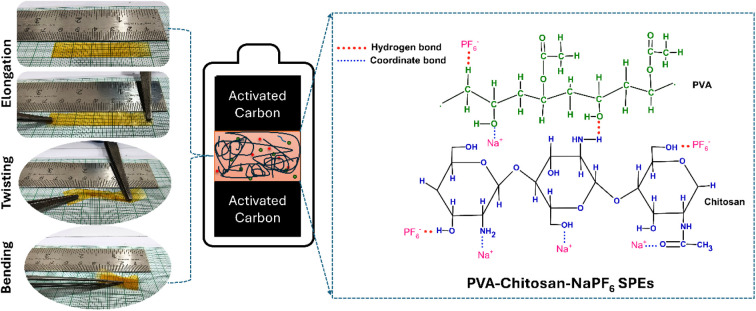

## 1 Introduction

Solid polymer electrolytes (SPEs) have emerged as valuable materials for use in modern electrochemical devices. They provide key benefits, such as enhanced safety, flexibility, and stability over traditional liquid electrolytes.^1^ These electrolytes are usually prepared by combining salts with polymers such as poly(ethylene oxide) or poly(ethylene imine).^2^ SPEs are used in various technologies including batteries, supercapacitors, fuel cells, and solar cells.^3,4^

SPEs have notable benefits for lithium-ion batteries, such as enhanced safety, flexibility, and minimized leakage risk.^5^ They are characterized by low flammability, ease of processing, and high thermal stability.^6^ Additionally, SPEs can prevent dendrite growth and provide improved mechanical strength.^7^ Despite these advantages, challenges like low ionic conductivity and limited contact at the electrode/electrolyte interface remain.^8^ Researchers have addressed this issue through strategies such as polymer blending, the use of plasticizers, fillers, and the incorporation of ionic liquids.^9^ Polymer blending improves SPE performance in several ways. It increases the amorphous regions in the polymer matrix, allowing ions to move more freely.^10^ The addition of nanoparticles can boost both mechanical strength and ionic conductivity simultaneously.^11^ Furthermore, combining different polymers enhances both the dimensional and chemical stability, as demonstrated in SPEEK/SPAES blends designed for fuel cell applications.^12^

Polyvinyl alcohol (PVA) and chitosan (CS) have been widely explored for biomedical applications because of their complementary properties. PVA is known for its flexibility, hydrophilicity, and robust mechanical strength,^13^ whereas CS is biocompatible, biodegradable, and has antibacterial properties.^14^ Blending PVA with CS enhances mechanical properties such as tensile strength and flexibility under both dry and wet conditions.^15^ Chitosan also improves PVA's biocompatibility, encouraging cell attachment and growth.^16,17^ PVA–chitosan blends exhibit better hydrophilicity, moisture regain, and water uptake compared to pure chitosan.^18^ The blends also demonstrate good compatibility and miscibility due to intermolecular hydrogen bonding.^19^ Recent research highlights that optimizing chitosan levels in PVA/chitosan hydrogels significantly boosts mechanical and viscoelastic properties, making these materials ideal for tissue engineering and drug delivery applications.^20^ Polymer blend electrolytes composed of PVA and CS offer a flexible and effective alternative for solid polymer electrolytes. These blends exhibit improved ionic conductivity, thermal stability, and mechanical strength in comparison to single-polymer systems.^21,22^ PVA/CS blends are well-suited for applications such as lithium-ion and proton-conducting electrolytes.^23,24^ Structural studies indicate that blending and the addition of salts increases the amorphous regions, which improves the ionic conductivity. These materials also exhibit excellent electrochemical stability and energy storage potential, making them ideal for energy storage devices.^25,26^

Sodium salts, such as NaPF_6_, are integral to the development of solid polymer electrolytes for sodium-ion batteries. When combined with polymers like PEO, PVA and PVDF–HFP, NaPF_6_ enhances ionic conductivity.^27,28^ For instance, the PVA–NaPF_6_ (with PVA : NaPF_6_ weight ratio of 60 : 40) exhibits the highest room-temperature ionic conductivity, reaching 3.65 × 10^−5^ S cm^−1^.^19^ NaPF_6_ is the preferred electrolyte salt because of its high ionic conductivity, electrochemical stability, and compatibility with electrode materials. Research indicates NaPF_6_-based electrolytes achieve ionic conductivities of 5–7 mS cm^−1^ at room temperature and remain stable up to 4.5 V *vs.* Na/Na^+^.^29^ Additionally, NaPF_6_ facilitates favorable surface film formation and enhances cathode material reversibility.^30^ Compared with NaClO_4_, NaPF_6_ offers superior Na^+^ desolvation kinetics and better interfacial stability. These properties make NaPF_6_ a strong candidate for sodium-ion battery electrolytes, combining high performance, stability, and conductivity.

NaPF_6_-based polymer electrolytes also enhance the performance of electric double-layer capacitors (EDLCs) by expanding their voltage window beyond 3.5 V, leading to improved energy density.^31^ Compared to traditional quaternary ammonium salts, NaPF_6_ offers superior rate performance and reduced ionic resistance in EDLCs.^32^ Furthermore, NaPF_6_-based electrolytes demonstrate comparable or better capacitance and cycling stability than LiPF_6_ and KPF_6_ counterparts, making them excellent options for high-performance EDLCs.^33^

**Fig. 1:**
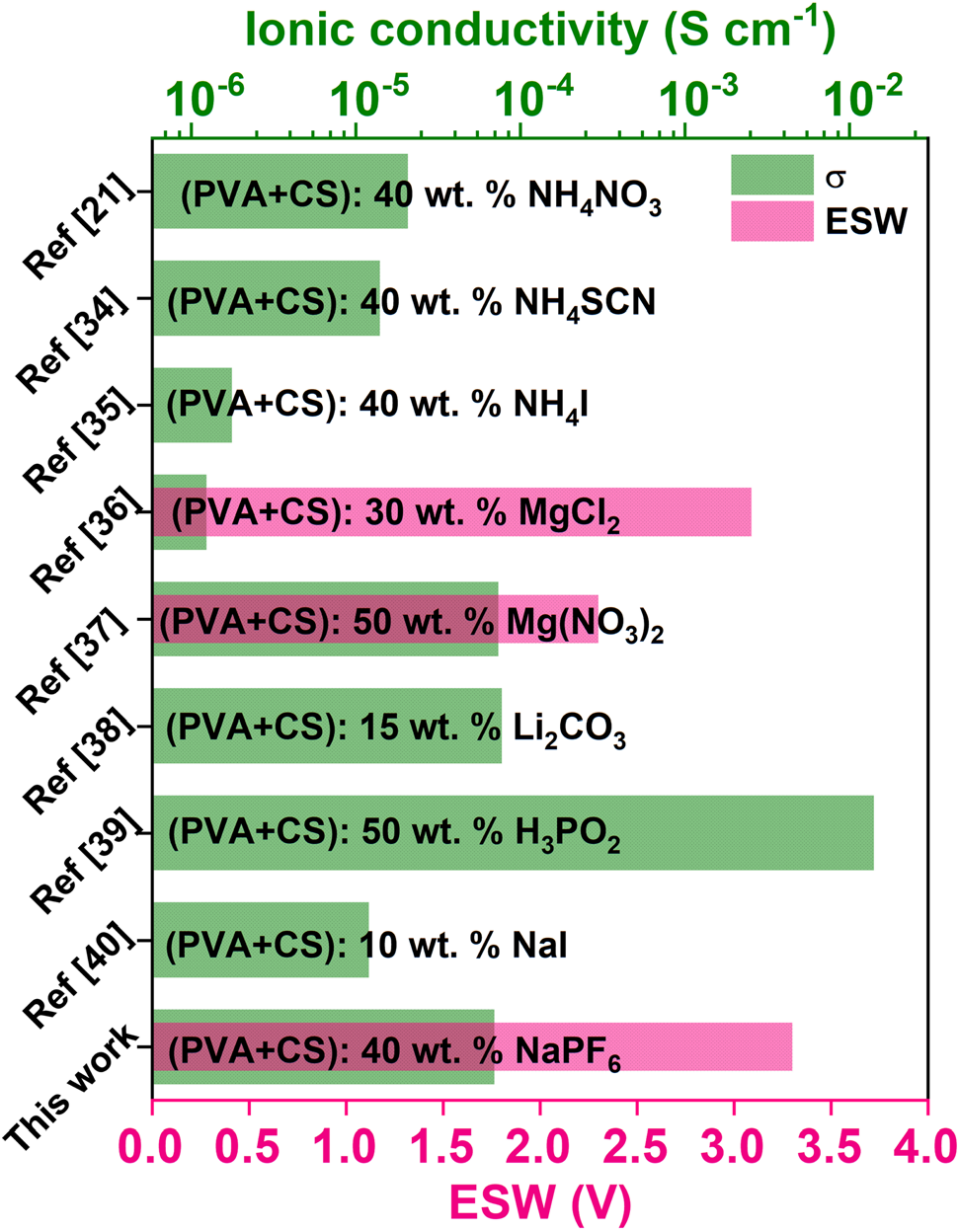
A bar graph depicting the peak conductivity along with electrochemical stability window of PVA/CS solid polymer electrolytes containing proton, lithium, magnesium and sodium ions.

Fig. 1 compiles earlier reports on PVA/CS electrolytes doped with protons, Li^+^, Mg^2+^ and Na^+^, pinpointing the best conductivities achieved so far along with electrochemical stability window (ESW).^21,34–40^ Achieving higher room temperature ionic conductivity is crucial for efficient ion transport and optimal battery performance. ESW is the voltage range within which the electrolyte remains stable without degradation. For PVA/CS-based SPEs, the ESW usually ranges from 2.0 V to 4.0 V, depending on the composition and inclusion of additives like plasticizers or ceramic fillers. A wider ESW enables the electrolyte to operate with electrodes at higher voltages, expanding its applications in energy storage systems. Balancing ionic conductivity and electrochemical stability is essential for optimizing SPE formulations based on the PVA/CS matrix, guiding material selection for specific battery technologies. This study focused on the development and investigation of a novel SPE system composed of a PVA and CS blend doped with NaPF_6_. A thorough review of the literature reveals that this specific combination has not been previously explored in terms of its structural, electrical, electrochemical, and mechanical properties. By addressing this gap, this research aims to provide a deeper understanding of the potential of this material for energy storage applications. The optimized SPE was further employed in the fabrication of an EDLC, with a detailed analysis of its discharge profiles. The results demonstrate the capability of the developed system to contribute to environmentally friendly energy-storage solutions. By utilizing biodegradable materials like PVA and CS, this work emphasizes the potential of sustainable SPEs to meet the growing need for eco-conscious technologies in advanced energy storage systems.

## 2 Experimental techniques

### 2.1 Materials used

Chitosan (CS; ≥75% deacetylated, 200 cPs viscosity in 1% solution; Loba Chemie), poly(vinyl alcohol) (PVA; *M*_w_ = 85 000 g mol^−1^, 86% hydrolysed; SD Fine Pvt Ltd), and sodium hexafluorophosphate (NaPF_6_; 167.95 g mol^−1^; Sigma-Aldrich) were combined to form the PVA/CS–NaPF_6_ host matrix. The 1 vol% acetic acid solvent was prepared by diluting glacial acetic acid (60.05 g mol^−1^; Merck Life Science, Bangalore) with deionized water. For EDLC electrode fabrication, activated carbon (AC; 1602 m^2^ g^−1^; Kuraray, Japan), carbon black (CB), polyvinylidene fluoride (PVDF), and *N*-methyl-2-pyrrolidone (NMP) were all obtained from Alfa Aesar (Mumbai). All reagents were used as received without further purification.

### 2.2 Solid polymer electrolyte preparation

Electrolyte films were fabricated by solution casting with 1 vol% acetic acid serving as the common solvent. PVA and CS were blended in a 60 : 40 wt% ratio, selected based on previous study,^41^ which demonstrated that this specific composition exhibits the highest degree of amorphous character among the tested blends. Increased amorphousness is advantageous because it facilitates more efficient ion transport within the polymer matrix, which is essential for achieving better electrochemical performance in supercapacitor applications. This blend was doped with varying NaPF_6_ contents as listed in Table 1. The doping level was restricted to 40 wt% because higher concentrations caused the films to become unstable and lose their free-standing nature. Also, beyond this concentration, the system transitions into a polymer-in-salt regime, where the polymer becomes the minority component and ion transport is primarily governed by diffusion through ion clusters rather than polymer segmental motion. The polymer–salt mixture received 100 mL of the acetic-acid solution and was magnetically stirred at 60 °C for 4 h to ensure complete dissolution of PVA. After the mixture cooled to room temperature, stirring continued for a further 24 h to promote thorough homogenization. The resulting solution was filtered to remove any residual CS particulates, poured into polyethylene Petri dishes, and dried at 40 °C for three days. Once fully solvent-free, the films were carefully peeled and transferred to a silica-gel vacuum desiccator for storage prior to characterization, a step that helps minimize moisture uptake.

**Table 1:** Sample designation

Designation	PVA/CS : NaPF_6_ (wt% : wt%)	PVA/CS (g)	NaPF_6_ (g)
PCX0	100 : 0	2.0	0
PCX5	95 : 5	1.9	0.1
PCX10	90 : 10	1.8	0.2
PCX15	85 : 15	1.7	0.3
PCX20	80 : 20	1.6	0.4
PCX25	75 : 25	1.5	0.5
PCX30	70 : 30	1.4	0.6
PCX35	65 : 35	1.3	0.7
PCX40	60 : 40	1.2	0.8

### 2.3 Fabrication of EDLC device

An EDLC device was fabricated to evaluate the practical applicability of the polymer electrolytes studied. Electrodes were prepared by dry blending AC and CB in an 85 : 15 weight ratio. PVDF binder solution was separately prepared by dissolving 1 g PVDF into 30 mL NMP. Subsequently, the binder solution was added incrementally to the AC–CB mixture and homogenized using a mortar and pestle, yielding a uniform slurry. Approximately 1 mg of the active material slurry was deposited onto stainless-steel electrodes using doctor blade method, which were then dried at 60 °C in an oven until completely dry. The EDLC stack was completed by inserting the highest-conductivity polymer electrolyte between the AC–CB electrodes.

### 2.4 Solid polymer electrolyte characterisation

#### 2.4.1 FTIR spectroscopy

FTIR spectroscopy was used to probe polymer–salt coordination in the SPE formulations. IR spectra were collected at ambient temperature in attenuated-total-reflectance (ATR) mode, eliminating the need for additional sample preparation while ensuring close contact. Transmittance data spanning 500–4000 cm^−1^ captured all relevant functional-group vibrations; shifts in these bands serve as fingerprints for changes in local bonding environments and confirm complexation between the polymer matrix and the dissolved salt.

#### 2.4.2 X-ray diffraction

XRD analysis of the SPE films was performed in reflection geometry to evaluate changes in crystallinity. Diffractograms were collected over 2*θ* = 5–90° with a scan rate of 2° min^−1^ and a step size of 0.02°. A Rigaku MiniFlex 600 diffractometer equipped with a monochromatic Cu-Kα source (*λ* = 1.54 Å, 40 kV, 15 mA) supplied the radiation. These parameters balance angular resolution with data-collection time, enabling reliable assessment of crystalline-to-amorphous phase evolution within the polymer matrix.

#### 2.4.3 Electrochemical impedance spectroscopy (EIS)

Dielectric spectra of the SPE films were recorded on a Hioki IM3570 impedance analyzer (Japan) over 298–373 K, with an AC frequency sweep from 50 Hz to 4.5 MHz. An excitation amplitude of 10 mV RMS ac voltage was kept low to maintain linearity when extracting the real (*Z*′) and imaginary (*Z*′′) impedance components. From these measurements, frequency and temperature-dependent dielectric constants and loss tangents were calculated, offering insight into dipolar polarization and charge-carrier dynamics within the polymer matrix. Ion dynamics within the SPE samples were investigated by converting the impedance data into various alternative formalisms. The mathematical relationships employed for these transformations are provided in detail in our earlier publication.^42^ This approach enabled a deeper analysis of relaxation processes, charge transport mechanisms, and interfacial phenomena governing the system's overall electrochemical performance.

The ionic conductivity of the SPE was calculated using the following equation:1
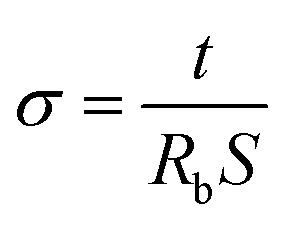
where *t* is the thickness of the SPE thin film, *S* is, the effective area of the electrodes, and *R*_b_ is the bulk resistance calculated from fitting Nyquist plot.

#### 2.4.4 Surface topographic studies

Surface morphology of the SPE films was first examined by scanning-electron microscopy (SEM); SEM micrographs were acquired at suitable magnifications and rescaled to accentuate key microstructural features. Complementary topographic information was gathered with an Innova-SPM atomic-force microscope (AFM) operating in tapping mode, employing p-doped Si cantilevers (spring constant 20–80 N m^−1^, resonance frequency 250–300 kHz) to record height images. The resulting data were processed to extract the root-mean-square surface roughness (*R*_q_), providing a quantitative measure of surface texture.

#### 2.4.5 Thermal studies

The thermal characteristics of the SPEs were investigated using differential scanning calorimetry (DSC) under a nitrogen atmosphere (flow rate: 40 mL min^−1^). Approximately 8 mg of sample material was placed in aluminum pans fitted with perforated lids and heated in a SHIMADZU DSC-60 PLUS instrument from 30 to 250 °C at a heating rate of 10 °C min^−1^. Thermogravimetric analysis (TGA) was carried out using a Hitachi STA7200 TGA-DTA instrument, where samples weighing between 5 and 8 mg were similarly placed into 40 μL aluminum pans equipped with perforated lids. Samples were heated from 30 °C to 500 °C at a rate of 10 °C min^−1^ under argon atmosphere. Conducting TGA under argon was essential to suppress oxidative effects, thereby providing accurate decomposition profiles of the materials.

#### 2.4.6 LSV and TNM studies

Linear-sweep voltammetry (LSV) was carried out on a CH Instruments CH600E potentiostat/galvanostat at a sweep rate of 10 mV s^−1^. Sodium–mercury amalgam (Na/Hg) electrodes served as both reference and counter, while a polished stainless-steel (SS) disc acted as the working electrode and the solid-polymer electrolyte (SPE) film was positioned between the SS and Na/Hg interfaces.

Total ionic transference number (*t*_ion_) was determined at 25 °C with a Keithley 2636B sourcemeter using a blocking-electrode stack (SS|SPE|SS). A constant bias of 100 mV was applied for 600 s, and current was logged every 0.01 s. The decay from the initial polarization current (*I*_0_) to the steady state value (*I*_ss_) was analyzed to extract (*t*_ion_), providing insight into the ionic *versus* electronic transport within the SPE films. The high-resolution time step ensures that the early transient regime which is critical for accurate *t*_ion_ evaluation is fully captured.

The total ionic transference number (*t*_ion_) was calculated using the following formula:2
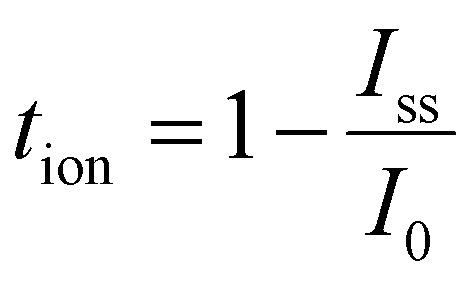


The ionic conductivity *σ*_ion_ was determined from the bulk conductivity and ion transference numbers using these equations:

3 *σ*_ion_ = *σ*_bulk_ × *t*_ion_

#### 2.4.7 Tensile testing

Mechanical characterization was performed on a 7200-series universal testing machine (Dak System Inc.). Three identical test samples were prepared following ASTM D880 and had gauge thicknesses between 0.10 and 0.20 mm. A constant cross-head velocity of 0.1 mm min^−1^ was applied to maintain a uniform strain rate, from which tensile strength, elongation at break and elastic modulus were determined. Variations among sample sets were evaluated with one-way ANOVA, and pairwise differences were resolved using Tukey's *post hoc* test at *α* = 0.05. This statistical protocol helps confirm that observed trends are not due to random variation.

### 2.5 EDLC characterisation

Electrochemical characterization was carried out on a Biologic SP-150e workstation, encompassing cyclic voltammetry (CV), galvanostatic charge–discharge (GCD), and electrochemical impedance spectroscopy (EIS). EIS spectra were recorded over 1 MHz to 0.01 Hz to capture the full frequency-dependent impedance response.

The specific capacitance obtained from CV data is calculated from,4
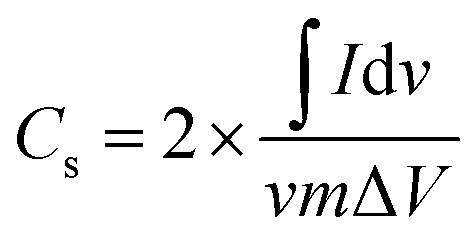


The specific capacitance (*C*_s_) obtained from GCD data is calculated from5
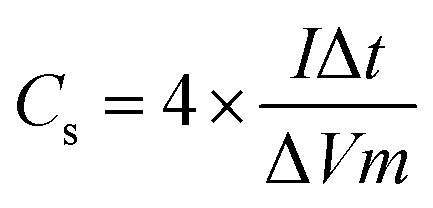


In this expression, *m* denotes the total mass of active material present on both electrodes. *I* is the discharge current (mA), Δ*t* is the discharge time (s) and Δ*V* is the effective discharge voltage. The factor of 4 converts the measured cell capacitance to a per-electrode basis—recognizing that an EDLC effectively comprises two capacitors (one on each electrode) in series. This normalization facilitates direct comparison of specific capacitance values with those reported for single-electrode systems.^43–47^

The energy and power densities^48^ were calculated using,6
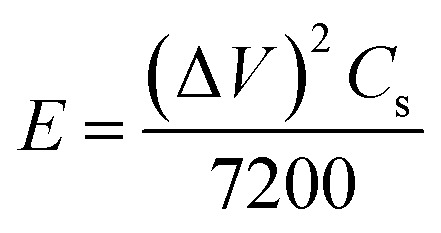
7
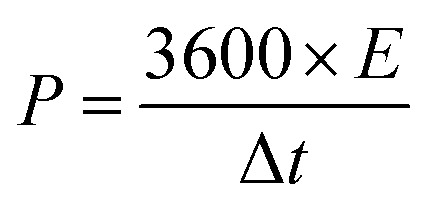


### 2.6 Results and discussion

#### 2.6.1 FTIR spectroscopy

FTIR spectroscopy is widely deployed to elucidate intermolecular interactions within SPEs. Shifts in the stretching or bending bands of key functional groups serve as fingerprints for such changes. Choosing an effective polymer host therefore hinges on chains rich in electron-donating hetero-atoms—most commonly oxygen and nitrogen, that can coordinate cations and promote salt dissociation, allowing the polymer to behave as a solid-state “solvent”.^49^ In the specific case of PVA/CS blends, previous studies have shown that intermolecular hydrogen bonds form between CS's amino and hydroxyl groups and the hydroxyl moieties of PVA, promoting miscibility and stabilizing the polymer network an effect that also tends to improve mechanical integrity.^50,51^

**Fig. 2:**
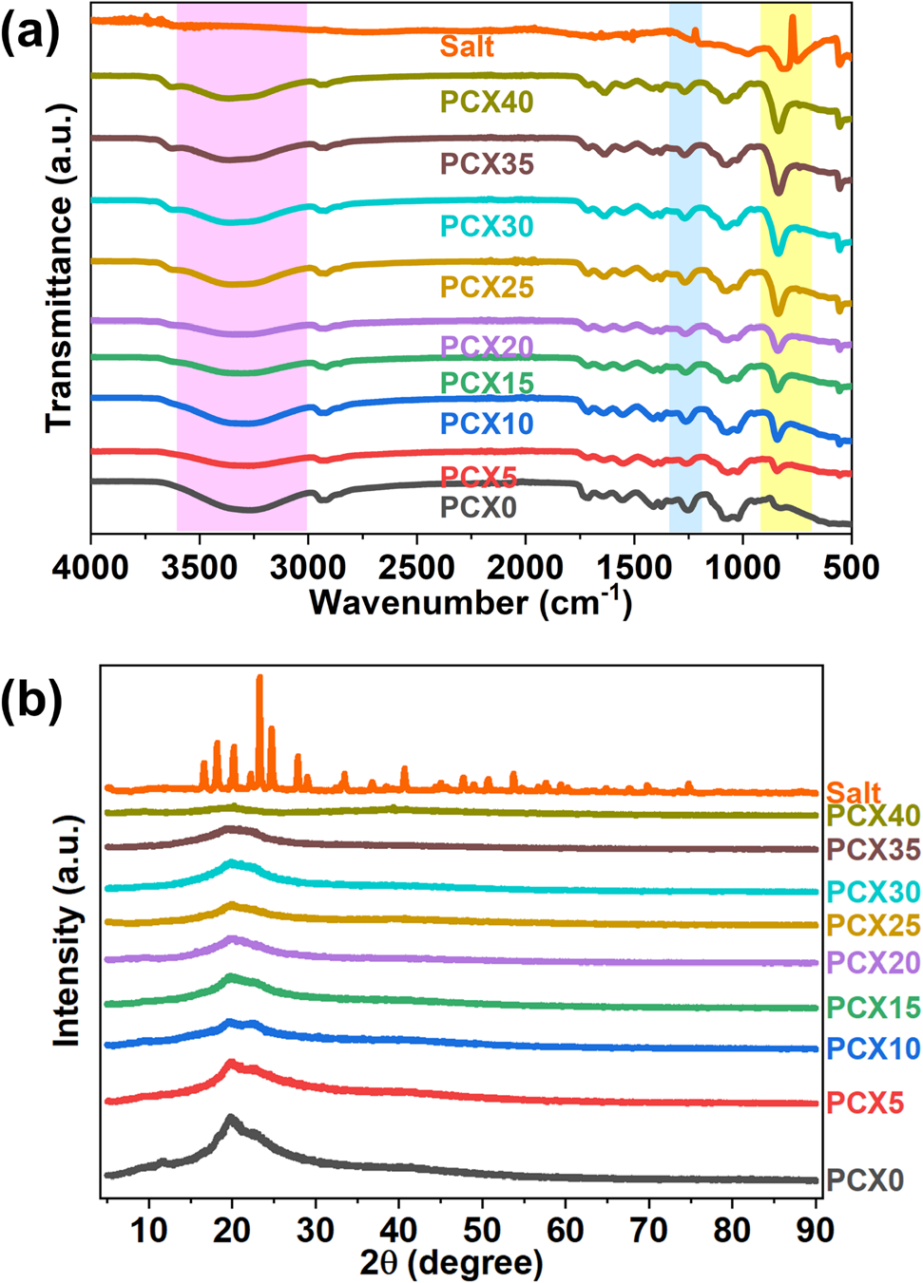
(a) FTIR spectra of PVA/CS–NaPF_6_ SPEs and NaPF_6_ along with (b) XRD spectra of PVA/CS–NaPF_6_ SPEs along with XRD spectra of NaPF_6_ salt.

Fig. 2(a), compares the FTIR profiles of the electrolyte films and the NaPF_6_ salt over the 400–4000 cm^−1^ window, while Table 2 lists the primary vibrational bands identified for the PVA/CS matrix.

**Table 2:** FTIR band assignments of PVA/CS blend and PVA/CS–NaPF_6_ SPEs

Bands	Samples along with wavenumbers associated with various functional groups (cm^−1^)								
	PCX0	PCX5	PCX10	PCX15	PCX20	PCX25	PCX30	PCX35	PCX40
–OH stretch	3262	3253	3274	3308	3348	3348	3367	3361	3366
–CH asymmetric stretch	2940	2943	2945	2948	2948	2946	2950	2946	2948
–CH symmetric stretch	2919	2923	2920	2917	2920	2919	2918	2919	2926
C=O stretch of PVA	1712	1714	1712	1712	1712	1712	1712	1712	1711
O=C–NHR carboxamide	1643	1641	1642	1642	1642	1641	1641	1632	1632
–NH_2_ bending	1555	1549	1553	1553	1548	1548	1548	1547	1547
–CH stretch	1412	1410	1413	1413	1413	1413	1414	1414	1414
–OH bending	1375	1379	1379	1379	1379	1379	1379	1379	1379
C–H wagging	1253	1262	1263	1264	1266	1266	1269	1270	1270
C–O bending	1074	1071	1073	1072	1073	1076	1075	1075	1077
C–O stretch of CS	1024	1024	—	1026	1026	1027	1026	1028	1028
Skeletal vibrations of PVA	946	—	—	—	—	949	949	948	947
CH rocking	826	846	842	841	840	839	837	836	839

The broad absorption centred between 3200 and 3550 cm^−1^ represents overlapping O–H and N–H stretching modes created by intra- and intermolecular hydrogen bonding in the PVA/CS matrix.^52^ In the NaPF_6_-doped films this composite band shifts from 3262 to 3366 cm^−1^, signalling coordination of Na^+^ ions with hydroxyl and/or amino sites. Earlier studies identify the –NH_2_ and –OH groups of chitosan as primary metal-ion anchoring centres; Na^+^ can bridge both groups simultaneously, disrupting the native –NH⋯OH hydrogen-bond network.^53,54^ A comparable effect occurs in PVA, where cation binding to –OH sites compete with existing inter-chain hydrogen bonds.^55^ The breaking and re-formation of these interactions suppress chain regularity, lower crystallinity, and thus hindering the reorganization of the polymer.

Furthermore, the introduction of salt induced significant shifts in multiple CH vibrational modes. Specifically, the CH rocking frequency shifted from 826 cm^−1^ to 839 cm^−1^, CH wagging moved from 1253 cm^−1^ to 1270 cm^−1^, CH symmetric stretching changed from 2919 cm^−1^ to 2926 cm^−1^ and CH asymmetric stretching shifted from 2940 cm^−1^ to 2948 cm^−1^. These observed variations suggest that the PF_6_^−^ anion is interacting with the CH groups of the PVA/CS matrix *via* hydrogen bonding. Previous research explored the interaction between PF_6_^−^ anions and the CH groups of the polymer, attributing it to weak hydrogen bonds and electrostatic forces. Specifically, studies indicate that the partially positive C–H hydrogen atoms can establish weak hydrogen bonds with the highly electronegative fluorine atoms in the PF_6_^−^ anion, with bond energies ranging from approximately 2.8 to 3.1 kcal mol^−1^.^56,57^ The PF_6_^−^ anion, being a large and symmetric anion, can exhibit weak electrostatic interactions with the hydrogen atoms of CH groups. These interactions are primarily due to the polarizability of the CH bonds, but they are not as strong as interactions with more electronegative atoms (like oxygen or nitrogen).^58^ While PF_6_^−^ is not a classic hydrogen bond acceptor like some other anions (*e.g.*, halides), there can be weak interactions between the fluorine atoms of PF_6_^−^ and the hydrogen atoms of the CH groups. However, these interactions are generally weaker than traditional hydrogen bonds.^59^ Also, the asymmetry observed in the broad band in the IR spectra of NaPF_6_ salt within the 800–900 cm^−1^ range suggests the coexistence of a PF_6_^−^ free anion peak, associated with the asymmetric P–F stretching band, and a peak corresponding to Na^+^⋯PF_6_^−^ contact ions.^60^ Consequently, changes in the salt concentration led to noticeable variations in the intensity of the CH rocking band within the 800–900 cm^−1^ region further corroborating polymer–salt interactions. Similar observations were reported by Yong *et al.*^19^ where NaPF_6_ complexed PVA showed variations in –C–H and –OH stretching IR vibrations. A schematic of the proposed interaction is provided in Fig. S1 (ESI file†).

#### 2.6.2 X-ray diffraction

The crystalline fraction of the blend membranes was quantified by XRD. Fig. 2(b) plots how increasing NaPF_6_ loading alters the diffraction profile. In well-crystallized polymers, diffraction peaks are sharp and intense, whereas amorphous regions give rise to broad, low-intensity halos.^61^ Individually, semi-crystalline PVA exhibits a wide halo centered at 2*θ* ≈ 19°,^62,63^ while chitosan shows reflections near 10°, 19°, and 22°.^64^ When the two polymers are combined, these signature peaks coalesce into a single broadened peak around 2*θ* ≈ 19.6°. The slight peak shift relative to the neat components is attributed to hydrogen bonding between the functional groups of PVA and CS, signaling full miscibility and a reduction in long-range order.^65,66^

Incorporating salt caused a reduction in the intensity of the characteristic peak at 2*θ* = 19.6°, as well as a decrease in the halo portion associated with the PVA/CS membrane. Additionally, as the concentration of salt increases, the intensity of the diffraction peak at 2*θ* = 11.52° diminishes, indicating a reduction in the crystalline phase of the SPEs.^67^ Incorporating salt into polymer electrolytes initially hinders crystallinity by disrupting the arrangement of polymer chains and fostering amorphous areas.^68^ Salt ions form complexes with polymer chains, which change their shape and prevent crystallization.^69^ The primary factor contributing to the reduction in the crystalline phase within this system is the coordination and hydrogen bonding interactions between the PVA–CS polymer matrix and the Na-salt, as evidenced by FTIR studies. This interaction results in a modification of the microstructural characteristics of the blend composites, shifting from a crystalline to a more amorphous state as the salt concentration increases from 5% to 40% by weight. The suppression of crystallinity generally enhances ionic conductivity by increasing the mobility of the polymer chains.^70^

The degree of crystallinity (*χ*) in the polymer films was determined using the Hermans and Weidinger method,^71^ which allows for the quantitative separation of crystalline and amorphous contributions from the X-ray diffraction pattern. This approach involves fitting the deconvoluted XRD spectrum (shown in Fig. S2, ESI file†) into distinct crystalline and amorphous regions. Based on this separation, the crystallinity was calculated using eqn (8):8
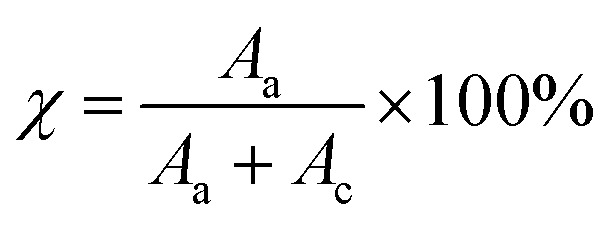
where *A*_c_ and *A*_a_ represent the integrated areas under the crystalline and amorphous peaks, respectively. This method provides a reliable estimate of crystallinity, particularly for semi-crystalline systems like PVA/CS blends. Details of the XRD peak-deconvolution procedure are described in our earlier work.^72,73^ The pristine PVA/CS blend displays a crystallinity of 14.94%, in good agreement with the value reported by previous report.^74^ Incremental doping with NaPF_6_ progressively suppresses the crystalline fraction, reaching a minimum of 3.59% at 40 wt% salt. The increase in amorphous content lowers the segmental-motion energy barrier, providing less hindered pathways for ion transport and, hence, higher ionic conductivity.^75^ On this basis analysis, the PCX40 film is the optimal conducting sample.

#### 2.6.3 Electrochemical impedance spectroscopy (EIS)

**Fig. 3:**
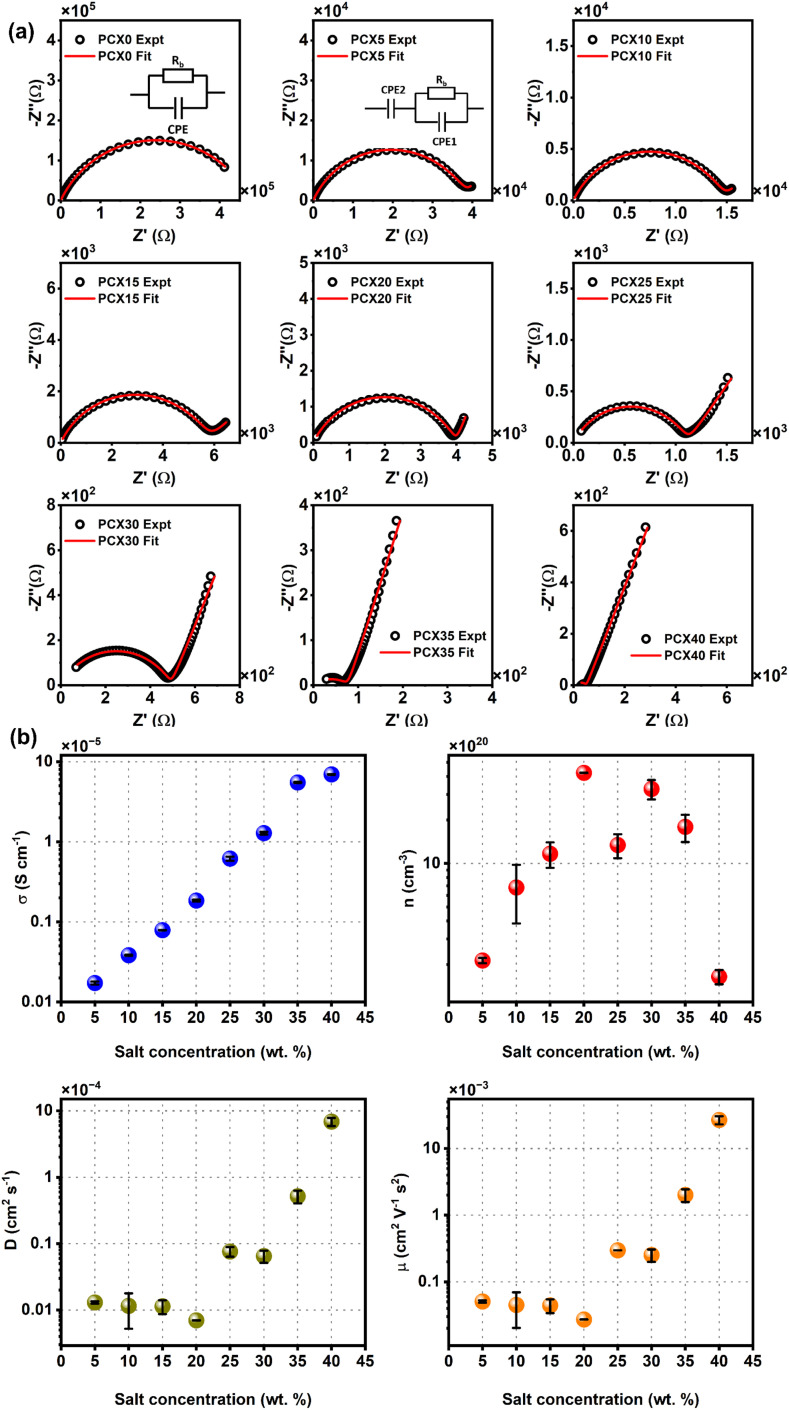
(a) Nyquist plot of PVA/CS–NaPF_6_ SPEs along with corresponding EEC which is used to fit it (inset) and (b) variation of ion transport properties as per S–G model along with bulk conductivity *σ* for PVA/CS–NaPF_6_ SPEs.

Fig. 3(a) shows Nyquist plots of the prepared SPEs. Nyquist plots of these SPEs typically feature a high-frequency semicircle and a low-frequency spike. For the undoped PVA/CS film (PCX0), only a depressed semicircle is seen, reflecting negligible ionic conduction. With NaPF_6_ addition (PCX5–PCX40), the semicircle in the high-frequency region becomes progressively compressed and a slanted line appears at lower frequencies, signaling enhanced ion transport at both the electrode–electrolyte interface and within the bulk. As salt concentration increases, the semicircle's diameter shrinks and the low-frequency spike lengthens, consistent with higher charge-carrier density and reduced charge-transfer resistance in the polymer matrix.

Fitting the Nyquist plot with a suitable electrical equivalent circuit (EEC) offers a straightforward yet powerful means of dissecting the system's electrochemical behavior. Through such EEC analyses, it is possible to directly determine the bulk resistance of the SPE, along with other components like interfacial resistances and capacitances, thus providing quantitative insights into ion transport and the interactions between electrodes and electrolytes.

Real SPE Nyquist patterns (Fig. 3(a)) rarely resemble an ideal Debye response. The high-frequency semicircle is depressed and the low-frequency spike tilts, because film thickness, morphology, and electrode roughness introduce heterogeneity.^76^ These non-idealities are handled by replacing the ideal capacitor in the equivalent circuit with a constant-phase element (CPE) whose impedance is 
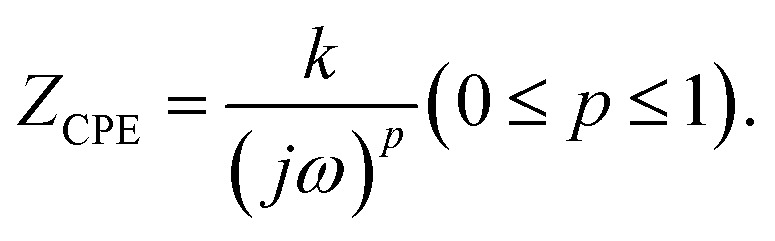
 At *p* = 0 the CPE behaves as a resistor (*k* = *R*), at *p* = 1 it reduces to a pure capacitor (*k*^−1^ = *C*), and intermediate *p* values stand for the distributed, “leaky” interfacial response.^77^ The specific EECs adopted for fitting are shown in the Fig. 3(a) insets.

The complex impedance equation corresponding to EEC of PCX0 is given by9
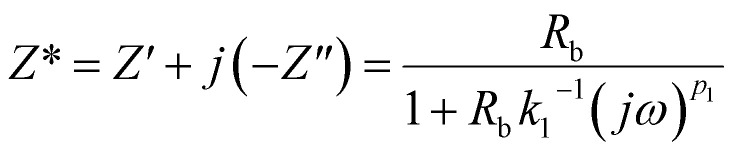


Simplifying eqn (9) into real (*Z*′) and imaginary parts (*Z*′′) yields eqn (10) and (11)10
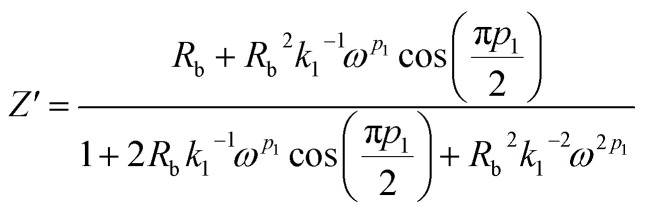
11
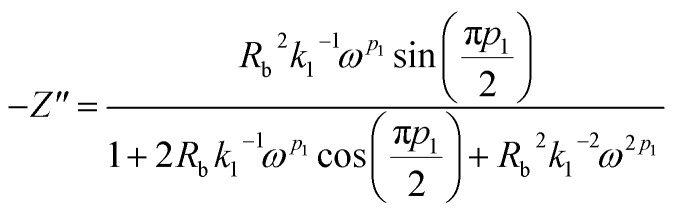


The complex impedance equation corresponding to EEC of PCX5–PCX40 is given as12
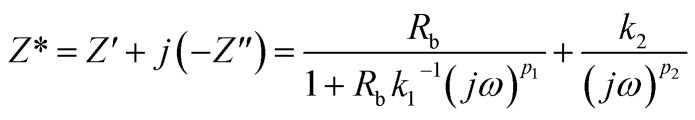


Simplifying eqn (12) into real (*Z*′) and imaginary parts (*Z*′′) yields eqn (13) and (14)13
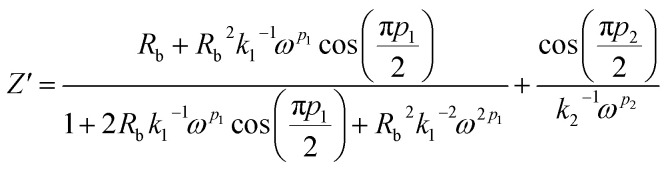
14
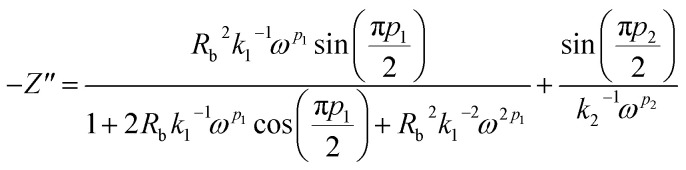


Non-linear least square fitting method was used to fit Nyquist plot data using the eqn (10), (11), (13) and (14) and the fitted parameter *R*_b_ and hence *σ* calculated from eqn (1) are listed in Table 3. According to the existing literature, a conductivity level of 10^−3^ to 10^−5^ S cm^−1^ is appropriate for use of electrolytes in energy storage systems. The conductivity of the SPEs increases (Table 3) with the salt concentration and reaches a maximum for PCX40 ((6.94 ± 0.04) × 10^−5^ S cm^−1^) which is three orders of magnitude higher than that of the pristine blend. The factors affecting the conductivity of the SPEs are now being discussed.

**Table 3:** Variation in bulk resistance *R*_b_, bulk conductivity *σ*, dc conductivity from ac conductivity *σ*_dc_, Jonscher power law fitting parameter *s*, conductivity relaxation time *τ*, along with regression coefficient *R*^2^ for PVA/CS–NaPF_6_ SPEs

Sample	*R*_b_ (Ω)	*σ* (S cm^−1^) at 25 °C	*σ*_dc_ (S cm^−1^)	*s*	(*τ*) (s)	*R* ^2^
PCX0	480 800 ± 7500	(1.43 ± 0.26) × 10^−8^	(4.71 ± 1.11) × 10^−8^	0.90 ± 0.01	—	0.99
PCX5	39 800 ± 1900	(1.72 ± 0.07) × 10^−7^	(2.10 ± 0.16) × 10^−7^	0.82 ± 0.01	—	0.99
PCX10	14 790 ± 200	(3.83 ± 0.05) × 10^−7^	(4.25 ± 0.17) × 10^−7^	0.79 ± 0.01	1.13 × 10^−3^	0.99
PCX15	5755 ± 29	(7.87 ± 0.03) × 10^−7^	(8.06 ± 0.20) × 10^−7^	0.79 ± 0.01	4.49 × 10^−4^	0.99
PCX20	4060 ± 110	(1.84 ± 0.04) × 10^−6^	(1.91 ± 0.02) × 10^−6^	0.75 ± 0.01	4.49 × 10^−4^	0.99
PCX25	1176 ± 70	(6.16 ± 0.35) × 10^−6^	(5.80 ± 0.04) × 10^−6^	0.77 ± 0.01	2.52 × 10^−4^	0.99
PCX30	508 ± 20	(1.28 ± 0.04) × 10^−5^	(1.25 ± 0.06) × 10^−5^	0.84 ± 0.01	3.99 × 10^−5^	0.99
PCX35	80.9 ± 1.1	(5.50 ± 0.07) × 10^−5^	(6.84 ± 0.45) × 10^−5^	1 ± 0.03	2.00 × 10^−5^	0.99
PCX40	52.46 ± 0.37	(6.94 ± 0.04) × 10^−5^	(7.21 ± 0.03) × 10^−5^	0.94 ± 0.02	2.24 × 10^−6^	0.99

To analyze the factors influencing conductivity, transport parameters were examined using the Schütt & Gerdes (S–G) model.^78^ According to the S–G model, the carrier concentration is given by15
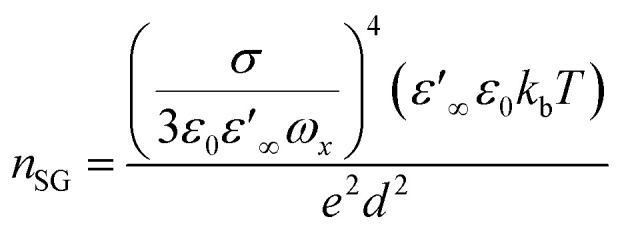


In eqn (15), *σ* is the DC conductivity, *k*_b_ is the Boltzmann constant, *ε*_0_ represents the permittivity in vacuum, and *d* signifies sample thickness. Here, *ε*_∞_ denotes the dielectric permittivity (real part) in the high-frequency region, *ω*_*x*_ stands for angular frequency, where *ε*′(*ω*_*x*_) = 10*ε*_∞_. Fig. 3(b) compares the Schütt–Gerdes transport metrics with the measured bulk conductivity. While the real permittivity *ε*′ follows the same trend as the charge-carrier density *n*, the dc conductivity *σ*_dc_ diverges, confirming that *σ*_dc_ is controlled by the combined effects of *n* and the carrier mobility *μ* rather than by *n* alone. At the highest salt loadings (35 and 40 wt%), *n* decreases, yet *μ* rises accordingly, offsetting the loss in carriers and shaping the overall conductivity profile.

#### 2.6.4 AC conductivity and tangent loss

Fig. S3(a) (ESI file†) depicts a log–log plot of the real component of the complex conductivity, *σ*_ac_*versus* frequency, revealing three characteristic regimes. At low frequencies, *σ*_ac_ declines because charge builds up at the electrode–electrolyte interface, generating a capacitive double layer whose internal field counteracts the applied field. This is followed by a frequency-independent plateau that represents the bulk or dc conductivity, *σ*_ac_. Beyond the plateau, *σ*_ac_ rises again in a high-frequency “dispersive” zone that captures local, frequency-activated ion-hopping processes sensitive to the polymer's chemical environment. The conductivity in this dispersive region obeys Jonscher's universal dynamic response, *σ*_ac_ = *A*(2π*f*)^*s*^, where *A* is a pre-exponential factor and the empirical exponent *s* usually lies between 0 and 1 (though values *s* > 1 have been reported) and varies with temperature. Mauritz linked smaller *s* values to more tortuous, less well-connected ion-transport pathways in hydrated Nafion,^79^ a concept that can be extended to other polymer-electrolyte systems. Hence, overall conductivity can be represented by

16 *σ*_ac_(*ω*) = *σ*_dc_ + *Aω*^*s*^

where *ω* is the angular frequency. The fit shows good agreement with the experimental data, indicating that the AC conductivity behavior follows a universal power law. The fitted parameters are listed in Table 3.

As the NaPF_6_ loading increases, the low-frequency tail in the *σ*_ac_ spectrum broadens, signalling a larger pool of mobile charge carriers. Fig. S3(b) (ESI file†) tracks the *σ*_ac_ spectra of optimized SPE membrane from 303 to 353 K where conductivity rises steadily with temperature, confirming thermally activated transport. Each curve shows a shallow maximum that shifts to higher frequency as *T* increases; beyond this point *σ*_ac_ drops sharply, the fall-off being steeper at the upper temperatures. This apparent “peak” originates from high-frequency instrumental noise rather than a genuine dielectric relaxation, and the elevated thermal energy amplifies conduction losses that restrict ion motion in this range.^80,81^ Because the spectrum lacks a true high-frequency dispersion branch, Jonscher's power-law formalism cannot be applied and the fit diverges.

Fig. S3(c) (ESI file†) plots the loss tangent (tan *δ*) against frequency for the NaPF_6_ doped SPEs. Every plot shows a single, asymmetric relaxation maximum that originates from conductivity relaxation within SPE films. As the salt fraction increases, this peak systematically moves to higher frequencies, signaling faster dipolar/ionic response.

The corresponding relaxation time was extracted using17
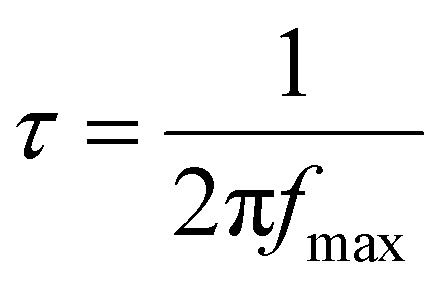
where *f*_max_ is the peak position; the calculated *τ*-values are listed in Table 3. Their downward trend confirms that greater NaPF_6_ loading accelerates ion dynamics across the polymer matrix.

#### 2.6.5 Dielectric analysis

Fig. S4(a) and (b) (ESI file†) displays room-temperature dielectric spectra for the prepared SPEs. Plotting *ε*′ and *ε*′′ on log–log scales clarify the separate fingerprints of each mechanism in the complex permittivity-ion/electron migration, interfacial charge build-up, and dipolar relaxation. Relaxation shows up as a step drop in *ε*′ accompanied by a peak in *ε*′′ as frequency increases, whereas conduction reveals itself through a low-frequency rise in *ε*′′. Purely ohmic transport leaves *ε*′ flat whereas non-ohmic or interfacial polarization, common in polymers,^82^ drives *ε*′ upward at the lowest frequencies. Accordingly, the spectra divide into an electrode-polarization zone followed by a dc-conduction zone, with no secondary relaxations visible at ambient conditions. Beyond about 10^6^ Hz the field changes too quickly for ionic motion, causing both *ε*′ and *ε*′′ to plummet. The pronounced low-frequency permittivity stems from electrical-double-layer formation at the electrode/electrolyte interface, consistent with a high concentration of mobile ions described by18
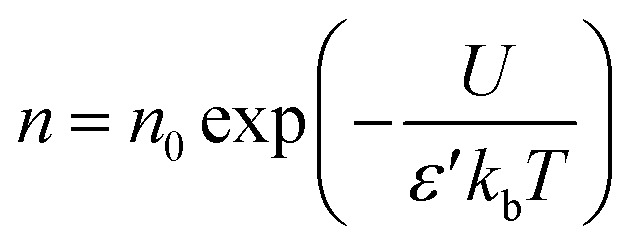
where *n* is the carrier density, *k*_b_ the Boltzmann constant, and *U* the salt-dissociation energy. PCX40 exhibits the largest low-frequency *ε*′, indicating the greatest free-ion population.

Fig. S4(c) and (d) (ESI file†) follow *ε*′ and *ε*′′ for PCX40 as temperature rises. Both increase because added thermal energy dissociates more Na^+^ and PF_6_^−^ ions, boosting the pool of mobile charges. Although dielectric spectroscopy can reveal secondary dipolar relaxations, none appear in *ε*′′(*f*) for PCX40 over the temperature range studied, implying that only the primary ion-related processes are active.^83^

#### 2.6.6 Surface studies

The surface morphology of electrolyte films is critical in defining texture, influencing the electrolyte/electrode interface, ion migration, and overall ionic conductivity. Fig. S5† displays the SEM micrographs of the current system at 1k× magnification. SEM images indicate that the samples' surfaces are uniform and smooth, showing no signs of phase separation.^84^ The XRD pattern's lack of crystalline peaks for the salts suggests no phase separation between the polymer and salt. Furthermore, the minute white spots in the micrographs were not from the undissociated salt, as they appeared in the pure polymer. The smooth surface background observed is indicative of the amorphous nature of the host polymer.^85^ Nevertheless, the samples demonstrated improved surface smoothness as the concentration of the dopant increased. The better surface morphology seen at elevated salt concentrations can be explained by the extensive distribution of salt throughout the polymer matrix, which leads to a more amorphous structure.^86,87^ As the salt content increased, the morphology became smoother, coinciding with an increase in ionic conductivity.^88^ The PCX40 membrane's EDAX spectrum (Fig. S5) (ESI file†) shows distinct C, O, and N peaks that are caused by the PVA–chitosan matrix in addition to distinct Na, P, and F signals that attest to the effective incorporation of NaPF_6_ salt.

Fig. S6 (ESI file†) displays the 2D and 3D AFM images for selected samples, with a scanning area of 5 μm × 5 μm. The undoped film (PCX0) had a rough surface with a roughness of 6.16 nm. Adding salt made the surface smoother, reducing roughness to 5.11 nm. This smoother surface may help improve contact between the electrode and electrolyte, which can boost device performance.^89^ This improvement in surface morphology is supported by the reduction in surface height variation of PCX40 from 32.2 nm to 21.3 nm.

#### 2.6.7 Thermal analysis

**Fig. 4:**
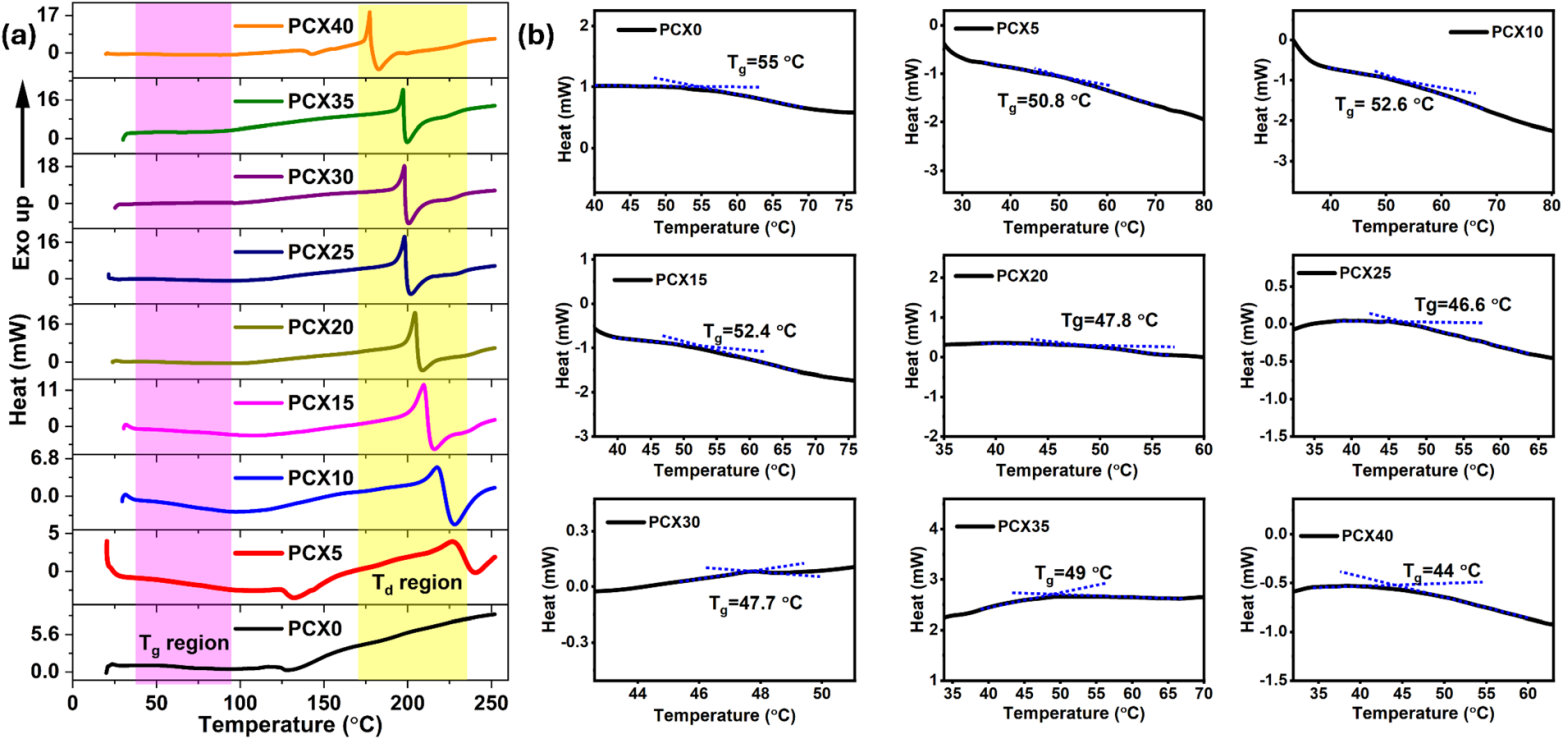
(a) DSC thermogram illustrating glass transition region and thermal decomposition regions and (b) glass transition temperature variation in PVA/CS–NaPF_6_ SPEs.

The DSC thermograms displayed in Fig. 4(a) for the current polymer system revealed two distinct temperature regions spanning from RT to 250 °C. A considerable endothermic peak emerged between RT and 100 °C, suggesting a glass-transition region. Additionally, there is a notable exothermic region beyond 150 °C, which corresponds to the decomposition area of the electrolytes. *T*_g_ is found out using the midpoint of the step transition (Fig. 4(b)).

All SPEs display a single glass-transition temperature (*T*_g_), confirming that polymer and salt are fully miscible. The non-linear shift of *T*_g_ and the lack of a melting peak shows that salt addition increases the amorphous fraction by fostering ion–dipole interactions between the ions and polar groups in the blend.^90^ The pronounced drop in *T*_g_ for the highest-conductivity sample, PCX40, compared with the undoped PCX0, illustrates the salt's plasticizing effect.^91^ TGA and DTG curves (Fig. S7†) reveal three mass-loss events for both doped and undoped SPEs. A 5–9% loss at 30–120 °C arises from residual acetic acid^92^ and loosely bound water attached to amine or hydroxyl sites.^93^ Between 180 and 237 °C the backbone undergoes chain scission and salt–polymer bonds break. The final stage, 350–500 °C, removes roughly 20% of the mass as the remaining char burns off. As shown in Table 4, the major decomposition temperature (*T*_d_) falls as dopant content rises, reflecting new salt–polymer complexes that alter the degradation pathway.^94^

**Table 4:** Variation of *T*_d_ and percentage mass loss for prepared SPEs

Sample	Major decomposition temperature *T*_d_ (°C)	% weight loss
PCX0	237	57
PCX5	229	61
PCX10	216	61
PCX15	205	65
PCX20	198	62
PCX25	192	70
PCX30	186	68
PCX35	181	72
PCX40	180	72

#### 2.6.8 LSV and TNM studies

**Fig. 5:**
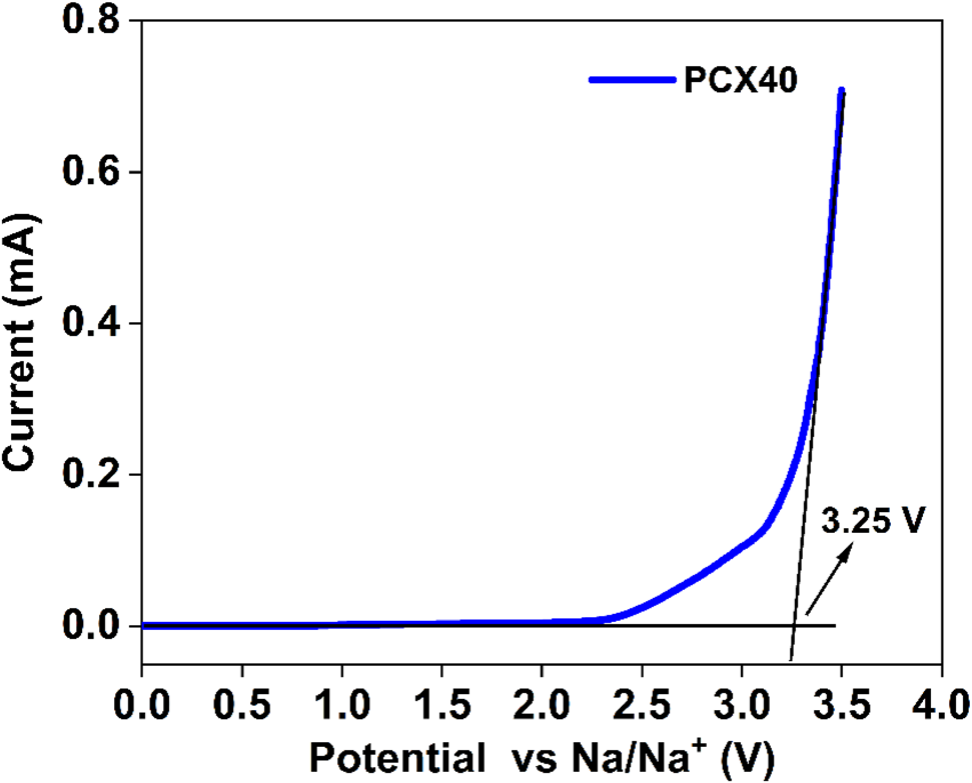
LSV plot of optimum conducting sample of PCX40 SPE.

LSV is a technique used to quantitatively describe the voltage stability range, also known as the electrochemical stability window (ESW), of SPEs. A wider ESW not only broadens the application scope of electrolytes but also enables the development of high-voltage, high-energy-density energy storage device.^95^ LSV plot for the optimum conducting sample is displayed in Fig. 5. The voltammograms exhibited a straight-line feature with minimal current, illustrating the ESW of the SPEs. The voltage range in which a significant increase in current occurs indicates an unsafe voltage region during cell operation. Notably, the PCX40 SPE exhibited an ESW of 3.25 V, which was greater than 3 V, making it suitable for realizing a prototype EDLC cell.

Fig. S8 (ESI file†) presents the chronoamperometry plot for the all the salt doped samples, obtained using Wagner's DC polarization method.^96^ The rapid decline in polarization current over time confirms the ionic conductivity of the fabricated electrolytes. The ion transport numbers along with ionic conductivity are tabulated in Table S1 (ESI file†). The *t*_ion_ values for these SPE films were found to be close to unity (>0.98), confirming that the electrolytes are ionic conductors, with minimal electronic contribution. This shows that ion transport governs overall conductivity. The results in this study align well with earlier studies.^97–99^

#### 2.6.9 Mechanical studies

**Fig. 6:**
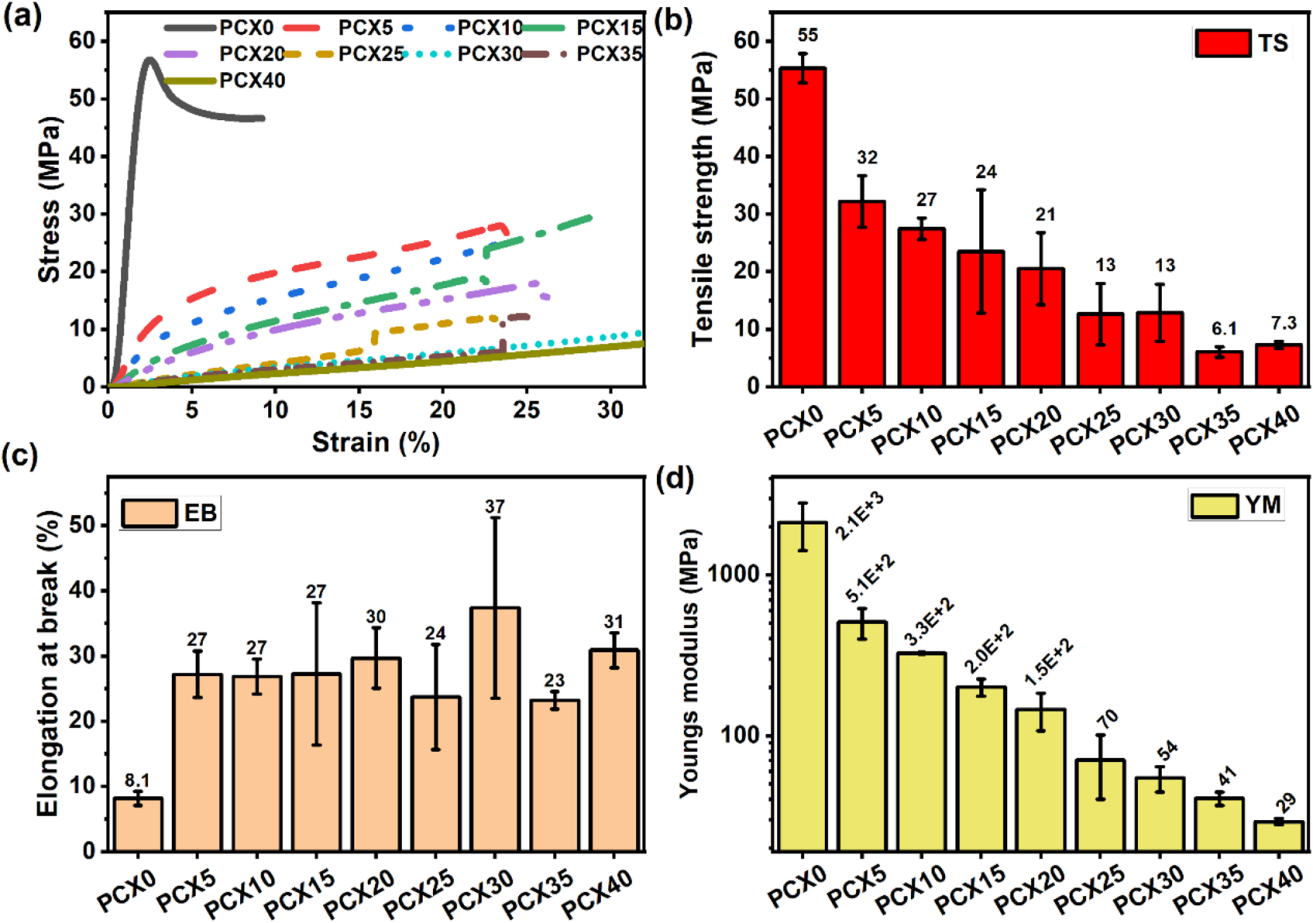
(a) Stress–strain graph and (b)–(d) variation of the mechanical parameters for PVA/CS–NaPF_6_ SPEs.

When used in energy storage devices, polymer electrolytes with excellent strength and toughness can accommodate electrode volume changes during charge–discharge cycles and effectively suppress dendrite formation, helping prevent short circuits and extend cycling lifespan.^100^The tensile test results for the synthesized electrolyte membranes are summarized in Fig. 6 and Table S2 (ESI file†). Typically, polymers with high crystallinity, strong cross-linking, or rigid molecular backbones display greater strength but limited flexibility which is reflected in a high Young's modulus and peak stress, alongside low elongation.^101^ As shown in the data, incorporating salt into the PVA–CS blend reduces both the Young's modulus and peak stress, while significantly increasing elongation at peak stress. This trend suggests a transition toward a more amorphous structure due to salt addition. The reduction in mechanical strength stems from the way Na^+^ ions interact with the polymer network.^102^ As the salt concentration increases, the PVA–CS blend evolves from a rigid, brittle material to one that is softer and more ductile. The PCX40 membrane, with 40 wt% NaPF_6_, achieves an elongation of over 29%, indicating enhanced flexibility and toughness.^103^

### 2.7 EDLC characterization

#### 2.7.1 Cyclic voltammetry

**Fig. 7:**
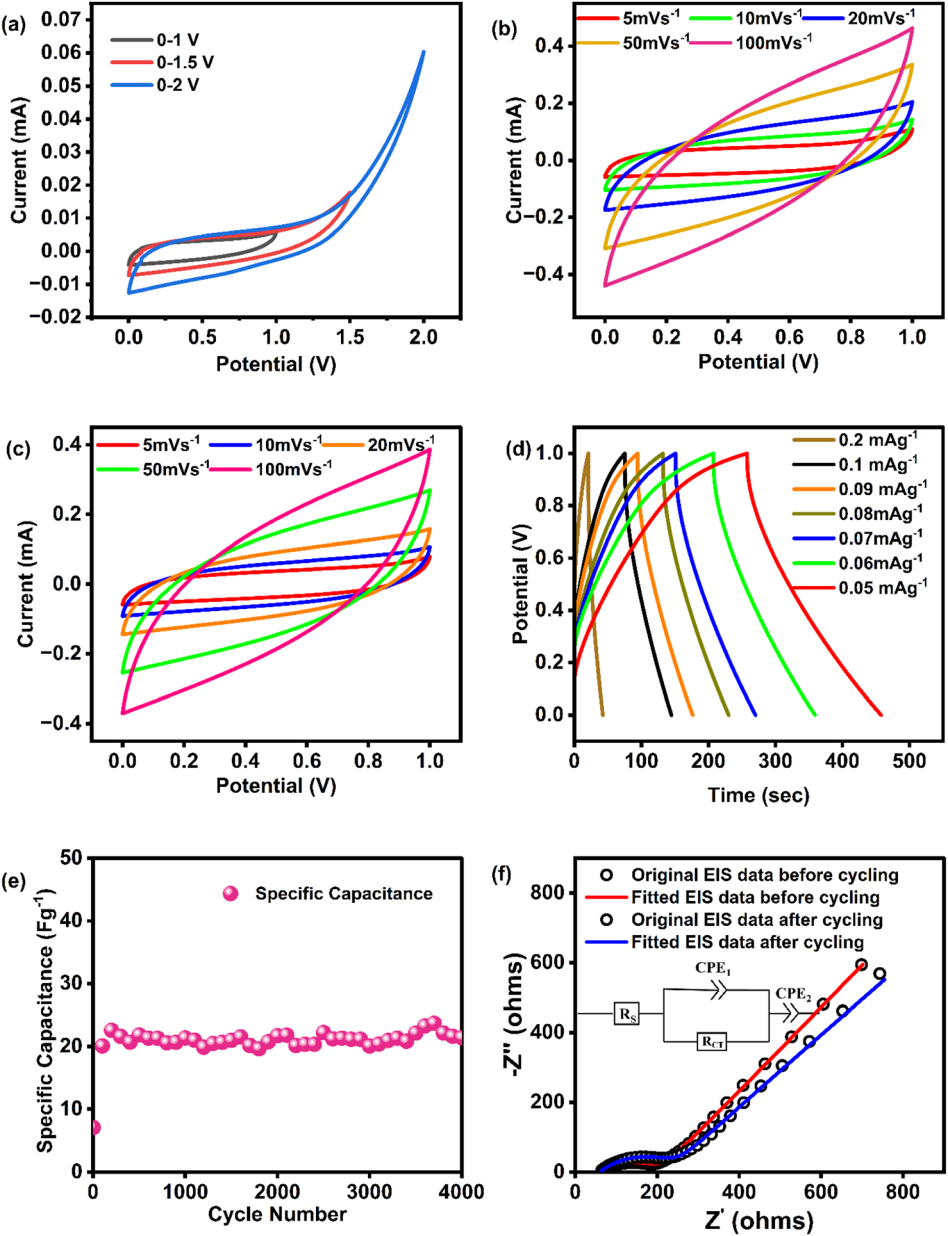
(a) CV profiles of EDLC device at various potential window (b) CV profiles of EDLC device at different scan rates before cycling (c) CV profiles after cycling (d) GCD of EDLC at different current densities (e) specific capacitance for 4000 cycles (f) Nyquist plot of EDLC before and after cycling.

The electrochemical performance of the assembled EDLC cell was investigated using cyclic voltammetry (CV) in a two-electrodes configuration to identify the charge-storage mechanism. Initially, CV measurements were recorded at a fixed scan rate of 5 mV s^−1^ across various voltage window, as illustrated in Fig. 7(a). The results indicate that the cell remains stable within the voltage range of 0–1 V. Beyond this range, distortion of the CV curves becomes apparent. Consequently, the optimal voltage window was identified as 0–1 V. After voltage optimization, CV measurements were repeated at scan rates ranging from 5–100 mV s^−1^ within this optimized voltage window, and the corresponding CV profiles are presented in Fig. 7(b). Within this voltage range, CV plots for the PCX40-based EDLC remain nearly rectangular and symmetric about the zero-current axis. The absence of redox peaks confirms that charge is stored purely through electric-double-layer formation rather than faradaic reactions.^104^ During charging, cations move toward the negative electrode and anions toward the positive one, while a strong electric field holds ions in the electrolyte and electrons on the carbon surface, producing the double layer where the device stores energy.^105^Fig. 7(b) illustrates that the CV plot diverges from an ideal rectangular shape due to voltage drops resulting from the internal resistance and porosity of the carbon electrodes, which vary with current. These profiles closely match those reported by Lavall *et al*.,^106^ indicating that charge and discharge at the electrode/polymer-electrolyte interface are nearly reversible. When the scan rate is low, ions adsorb at the interface, creating a stable double-layer charge, which accounts for the nearly perfect plateau observed. This happens because a thick diffuse layer develops, decreasing ohmic resistance. In contrast, at a high scan rate, the diffuse layer is thinner, leading to reduced capacitance.^105^

Specific capacitance (*C*_s_), calculated from the CV data with eqn (4) and summarized in Table 5, is highest at low scan rates and falls steadily as the scan rate rises. Slow scans allow ions enough time to build a thick diffuse layer and a stable electric double layer, producing a broad, nearly flat current plateau and lower ohmic resistance. At higher scan rates, ion transport toward the electrode surface occurs more rapidly, limiting the time available for complete double-layer formation. As a result, the development of a well-defined electric double layer becomes less efficient under these conditions.^107^ Cyclic voltammetry was also employed to assess the device's durability through repeated cycling. After 4000 charge–discharge cycles (Fig. 7(c)), the CV plot shows only a minor drop in *C*_s_ (Table 5), confirming that the EDLC retains its performance over prolonged cycling.

**Table 5:** *C*_s_ using CV profiles at different scan rates

Scan rate (mV s^−1^)	*C*_s_ before cycling (F g^−1^)	*C*_s_ after cycling (F g^−1^)
100	6.4	5.8
50	10.4	8.8
20	18	14.9
10	24.6	18
5	28	24

#### 2.7.2 Charge–discharge profiles of the EDLC

Galvanostatic cycling was employed to evaluate the EDLC, with its charge–discharge trace presented in Fig. 7(d). As illustrated in the plot, the GCD curves exhibit a triangular profile, characteristic of capacitive behavior. Notably, each curve shows a two-step feature. The initial abrupt voltage drop at the beginning of discharge corresponds to the internal resistance (*iR*_drop_), which is likely influenced by the relatively high equivalent series resistance (ESR) associated with the disordered structure of the activated carbon (AC) electrode. Following this, the discharge segment transitions into a linear slope, further confirming the capacitive nature of charge storage in the device. The *C*_s_ values were extracted from these curves *via*eqn (5) and tabulated in Table 6.

**Table 6:** Specific capacitance, energy, and power density calculated using GCD analysis

Current density (mA g^−1^)	Specific capacitance (F g^−1^)	Energy density (W h kg^−1^)	Power density (W kg^−1^)
0.05	42.65	5.3	95
0.06	38.20	4.7	114
0.07	35.48	4.4	133
0.08	34.06	4.0	147
0.09	32.43	3.8	162
0.1	31.50	3.4	178
0.2	25.27	2.0	304

#### 2.7.3 Cycling stability and EIS analysis

Fig. 7(e) shows, the PCX40-based device preserved its performance throughout, confirming outstanding cycling stability and efficiency. Fig. 7(f) shows the Nyquist response of the PCX40/AC–CB cell before and after cycling for 4000 charge–discharge cycles. The plot depicts a high-frequency semicircle and a low-frequency spike. These features separate into (i) bulk electrolyte resistance given by the high-frequency *x*-intercept, (ii) charge-transfer resistance at the electrode/electrolyte interface producing the semicircle, and (iii) ion diffusion inside electrode pores responsible for the low frequency spike. The equivalent circuit (inset) consists of a series ohmic resistance (*R*_s_) in series with a parallel combination of charge-transfer resistance (*R*_ct_) and constant-phase element CPE1, followed by a second constant-phase element CPE2. The first and second real-axis intercepts mark *R*_s_ (contacts and leads) and *R*_ct_, respectively. Impedance spectrum taken before and after 4000 cycles shows only small rise in *R*_s_ and *R*_ct_, indicating fast ion migration and negligible degradation. The data in Table 7 confirm that the EDLC retains its original electrochemical properties during extended cycling. The cell was subjected to 4000 charge–discharge cycles at 0.2 mA g^−1^.

**Table 7:** Estimated values of the equivalent circuit components in the model used for fitting the EIS plot before and after cycling

Element	Cycling	Values
*R*_s_	Before	59.2 Ω
	After	59.1 Ω
R_ct_	Before	153.4 Ω
	After	211.3 Ω

#### 2.7.4 Efficiency and ESR

**Fig. 8:**
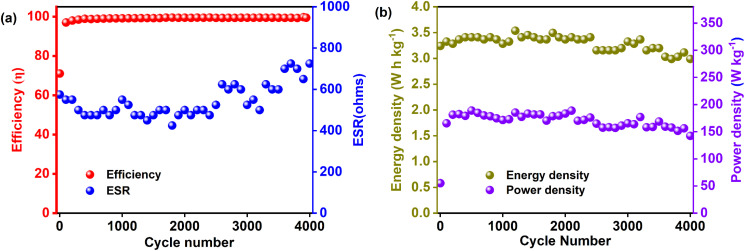
(a) Efficiency and ESR and (b) energy and power density values of PCX40-based EDLC over 4000 charge–discharge cycles.

The coulombic efficiency, *η*, quantifies EDLC stability and is defined as:19
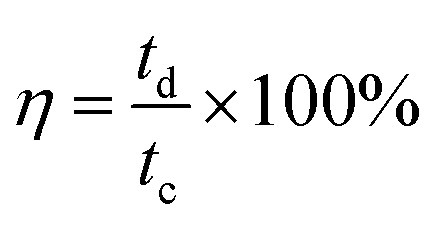
where *t*_d_ and *t*_c_ are the discharge and charge durations, respectively. As shown in Fig. 8(a), *η* remains essentially 100% unchanged over 4000 cycles, indicating a robust, reliable electrode–electrolyte interface throughout cycling.^108^ Equally important is the equivalent series resistance (ESR),^107^ which depends on the intrinsic resistance of the electrode material, the electrolyte, the current collector, and the electrode–electrolyte boundary.^104^ ESR is calculated from the voltage drop (*iR*_drop_) during galvanostatic cycling by the following equation:20
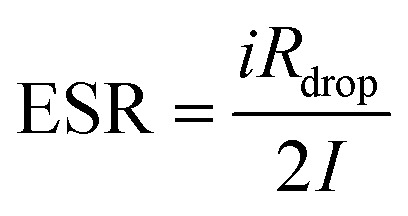


Fig. 8(a) illustrates that ESR starts at about 575 Ω in the first cycle and then settles around 700 Ω by cycle 4000. This gradual increase in *iR*_drop_ and hence ESR can stem from slight degradation of the polymer electrolyte, whose conductivity here is about 10^−5^ S cm^−1^.^109^ Enhancing electrolyte conductivity with plasticizers or more mobile salts can lower ESR. The values observed align well with those reported for other EDLC systems.^109–111^

#### 2.7.5 Energy and power density

Energy and power densities, calculated *via*eqn (6) and (7) and plotted over 4000 cycles at 0.2 mA g^−1^ (Fig. 8(b)), show remarkable steadiness. The energy density remains unchanged throughout cycling, indicating that ions continue to surmount a similar energy barrier without significant clustering.^112^ Likewise, the power density of 308 W kg^−1^ in the first cycle—displays only negligible drift over the full 4000 cycles, a behavior consistent with prior reports.^113^ A comparative analysis of the EDLC parameters obtained in this study with those reported in earlier works is summarized in Table 8. This comparison highlights the relative performance and potential advantages of the present system.

**Table 8:** Parameters of the fabricated EDLC of optimum sample in this report compared with previously reported systems

Electrolyte system	Specific capacitance (F g^−1^)	Energy density (W h kg^−1^)	Power density (W kg^−1^)	Nature of electrolyte	Reference
Chitosan–PEO–LiClO_4_	6.88 @ 0.5 mA cm^−2^	1.07	321	Solid electrolyte	114
PVA–CS–NaSCN–glycerol	55.5 @ 20 mV s^−1^	12.96	2054	Plasticized electrolyte	115
CS : MgCl_2_–glycerol	50 @ 10 mV s^−1^	13.1	550	Plasticized electrolyte	116
PVA–CS–MgCl_2_	5.80 @ 5 mV s^−1^	—	—	Solid electrolyte	37
PVA–CS–NaClO_4_·H_2_O	38.45 @ 5 mV s^−1^	2.69	97	Solid electrolyte	117
PVA–CS–PEG–Mg(NO_3_)_2_	4.24 @ 5 mV s^−1^	1.89	62.42	Plasticized electrolyte	118
PVA–CS–NaPF_6_	28 @ 5 mV s^−1^	5.3	95	Solid electrolyte	Present work

## 3 Conclusions

SPEs based on CS and PVA were synthesized by incorporating varying amounts of NaPF_6_. The sample with the highest ionic conductivity was subsequently utilized to fabricate an EDLC. XRD analysis confirmed the predominantly amorphous nature of the SPEs, while FTIR spectroscopy underscored the strong interactions between the polymer matrix and the salt dopant. Furthermore, the relatively smooth surface morphology suggests good compatibility between the salt and the polymer phases. EEC model was applied to the prepared samples to obtain a comprehensive understanding of their electrical characteristics. The electrochemical performance of the EDLC cell was assessed under ambient conditions *via* impedance spectroscopy, CV, and GCD. The characteristic “leaf-shaped” CV curves, with no observable redox peaks, confirmed the non-faradaic, electric double-layer mechanism at the electrode–electrolyte interface. At a current density of 0.05 A g^−1^, the specific capacitance, energy density, and power density for the optimum electrolyte-based cell reached 42.65 F g^−1^, 5.3 W h kg^−1^, and 95 kW kg^−1^, respectively. Moreover, an EDLC with a polymer electrolyte containing 40 wt% NaPF_6_ retained good stability over 4000 charge–discharge cycles. These findings highlight the potential of bio-polymer blend electrolytes for constructing high-performance EDLC devices, with further conductivity enhancements achievable using various additives.

## Consent for publication

The authors hereby consent to publication of the present research work in this journal, if selected for publication.

## Author contributions

Vipin Cyriac: conceptualization, data curation, formal analysis, investigation, methodology, writing – original draft. Ismayil: methodology, resources, supervision, validation, writing – review & editing. Kuldeep Mishra: resources, validation. Ankitha Rao: investigation, formal analysis. Riyadh Abdekadir Khellouf: formal analysis, validation. Saraswati P. Masti: formal analysis, resources, validation. I. M. Noor: validation, formal analysis, writing – review & editing.

## Conflicts of interest

The authors declare that they have no known competing financial interests or personal relationships that could have appeared to influence the work reported in this paper.

## Supplementary Material

RA-015-D5RA02897C-s001

## Data Availability

Data will be made available on request upon contacting the corresponding author.
