# Supplementary material for: Sodium hexafluorophosphate mediated enhancement of electrical and electrochemical properties of poly(vinyl alcohol)–chitosan solid polymer electrolytes for EDLCs
Source: RSC Adv. 2025 Jul 11;15(30):24350–66. doi: 10.1039/d5ra02897c (PMC12247050; doi:10.1039/d5ra02897c)
Supplement: RA-015-D5RA02897C-s001 [file RA-015-D5RA02897C-s001.pdf]

Supplementary Information (SI) for RSC Advances.
This journal is © The Royal Society of Chemistry 2025

Supplementary material

Sodium hexafluorophosphate mediated enhancement of electrical and electrochemical properties of poly (vinyl alcohol)–chitosan solid polymer electrolytes for EDLCs

Vipin Cyriac[a], Ismayil[a*], Kuldeep Mishra[b], Ankitha Rao[c], Riyadh Abdekadir Khellouf[d], Saraswati P Masti[e], I. M Noor[f,g],

[b]*Department of Physics, Manipal Institute of Technology, Manipal Academy of Higher Education, Manipal 576104, Karnataka, India*

[b]*Symbiosis Institute of Technology (SIT), Symbiosis International (Deemed university) (SIU), Pune 412115, Maharashtra, India*

[c]*Department of Electronics and Communication, Manipal Institute of Technology, Manipal Academy of Higher Education, Manipal 576104, Karnataka, India*

[d]*Centre of Polymer Systems, University Institute, Tomas Bata University in Zlin, Tr. T. Bati 5678, 760 01 Zlin, Czech Republic*

[e]*Department of Chemistry, Karnataka University's Karnataka Science College, Dharwad, Karnataka 580001, India*

[f]*Ionic Materials and Energy Devices Laboratory, Physics Department, Faculty of Science, Universiti Putra Malaysia, 43400 UPM Serdang, Selangor Darul Ehsan, Malaysia*

[g]*Physics Division, Centre for Foundation Studies in Science of Universiti Putra Malaysia, Universiti Putra Malaysia, 43400 Serdang, Selangor Darul Ehsan, Malaysia*

[*]*Corresponding Author e-mail: ismayil.mit@manipal.edu, ismayil.486@gmail.com,*
Tel: +91 98454 97546

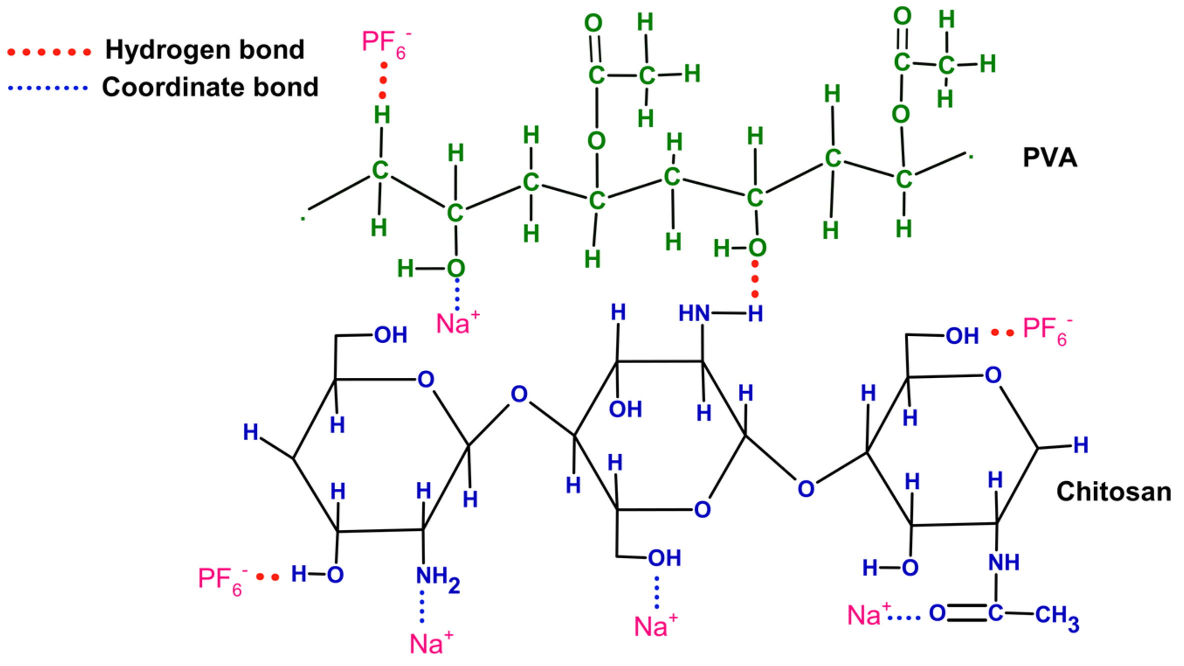

Figure S1: Interaction scheme of dopant with PVA and CS in PVA/CS-$NaPF_6$

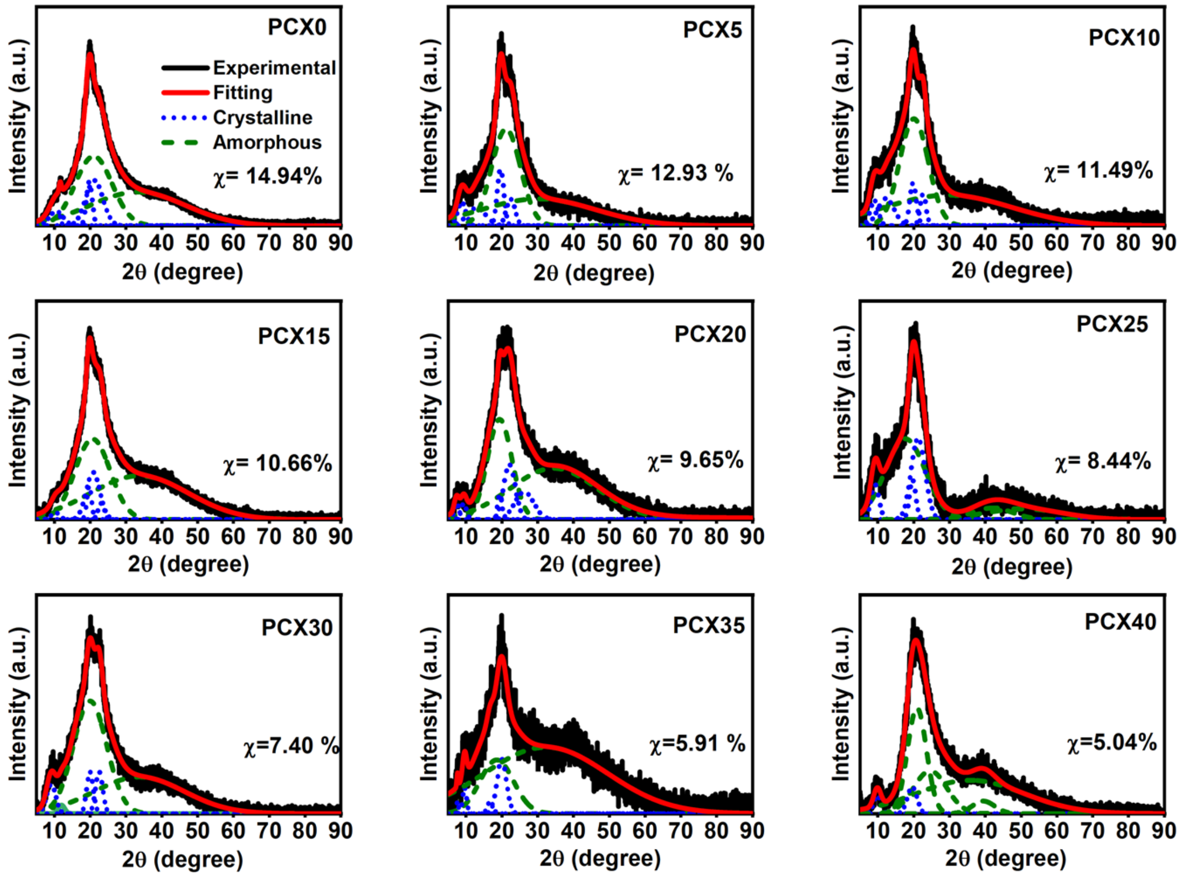

Figure S2: Deconvoluted XRD pattern of PVA/CS-$NaPF_6$ SPEs

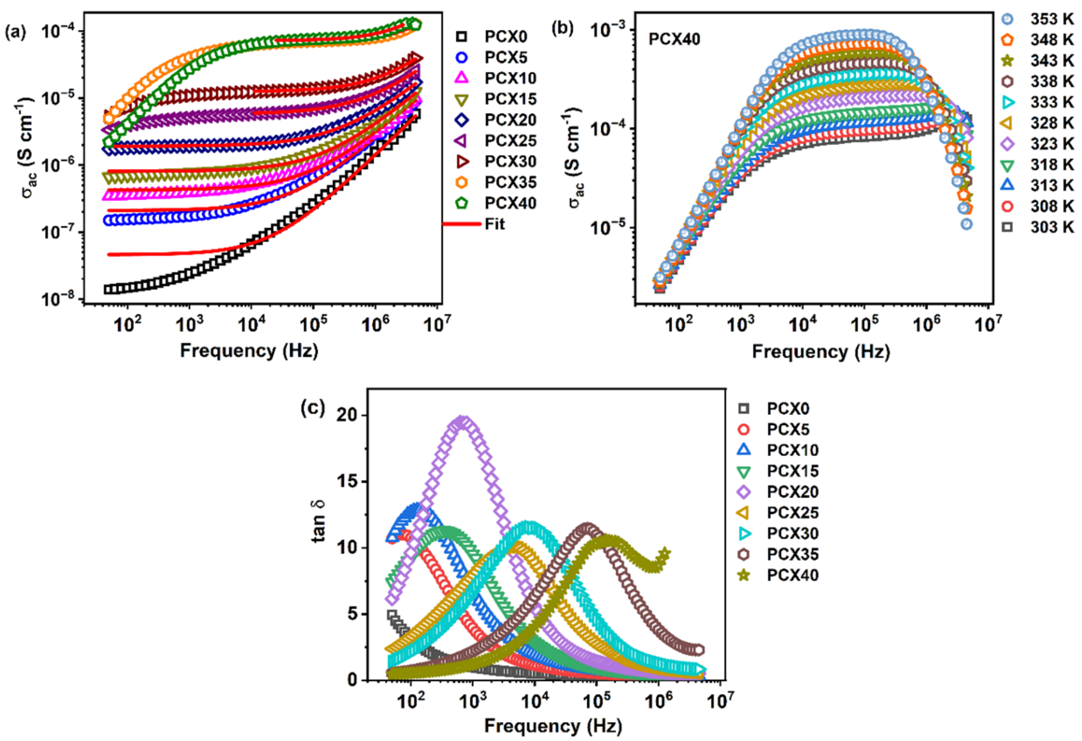

Figure S3: (a) AC conductivity of the PVA/CS-$NaPF_6$ SPEs at room temperature (JPL Fit is given as red solid line) (b) AC conductivity of optimum conductivity sample PCX40 for various elevated temperatures and (c) tangent loss plot for PVA/CS-$NaPF_6$ SPEs at room temperature.

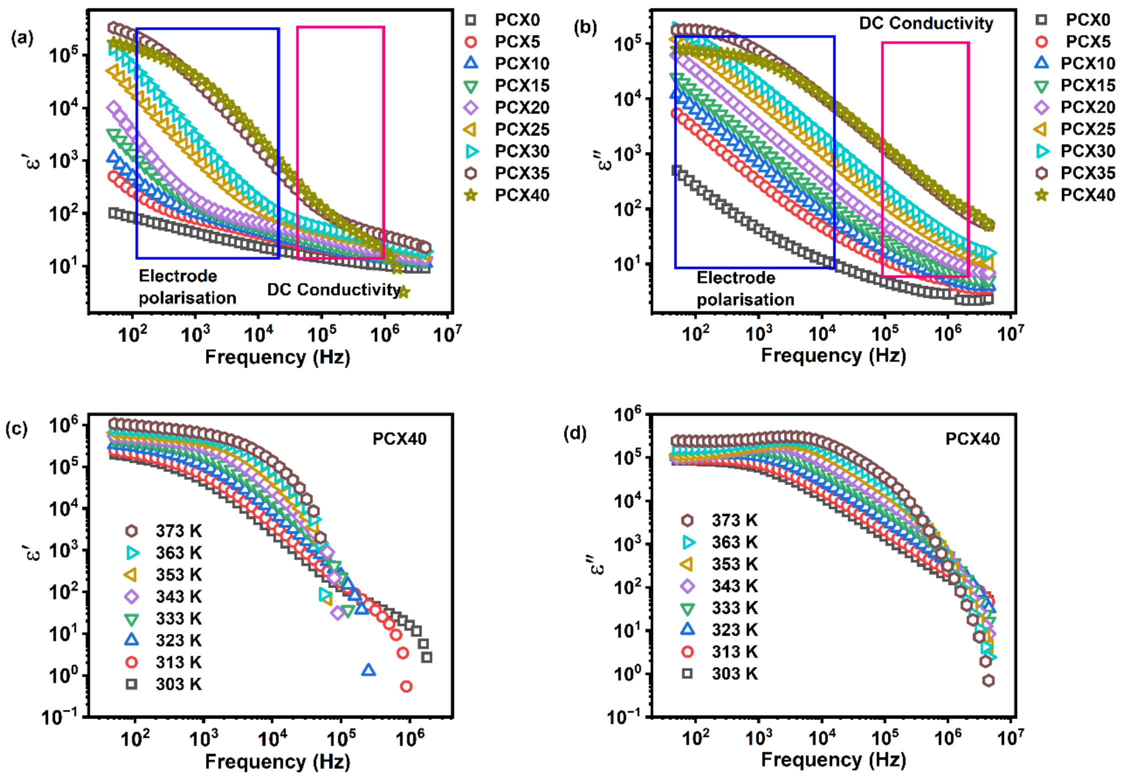

Figure S4: Variation in (a) ϵ', (b) ϵ'' for PVA/CS-$NaPF_6$ SPEs, (c)ϵ' and (d) ϵ'' for elevated temperature for optimum conductivity sample PCX40.

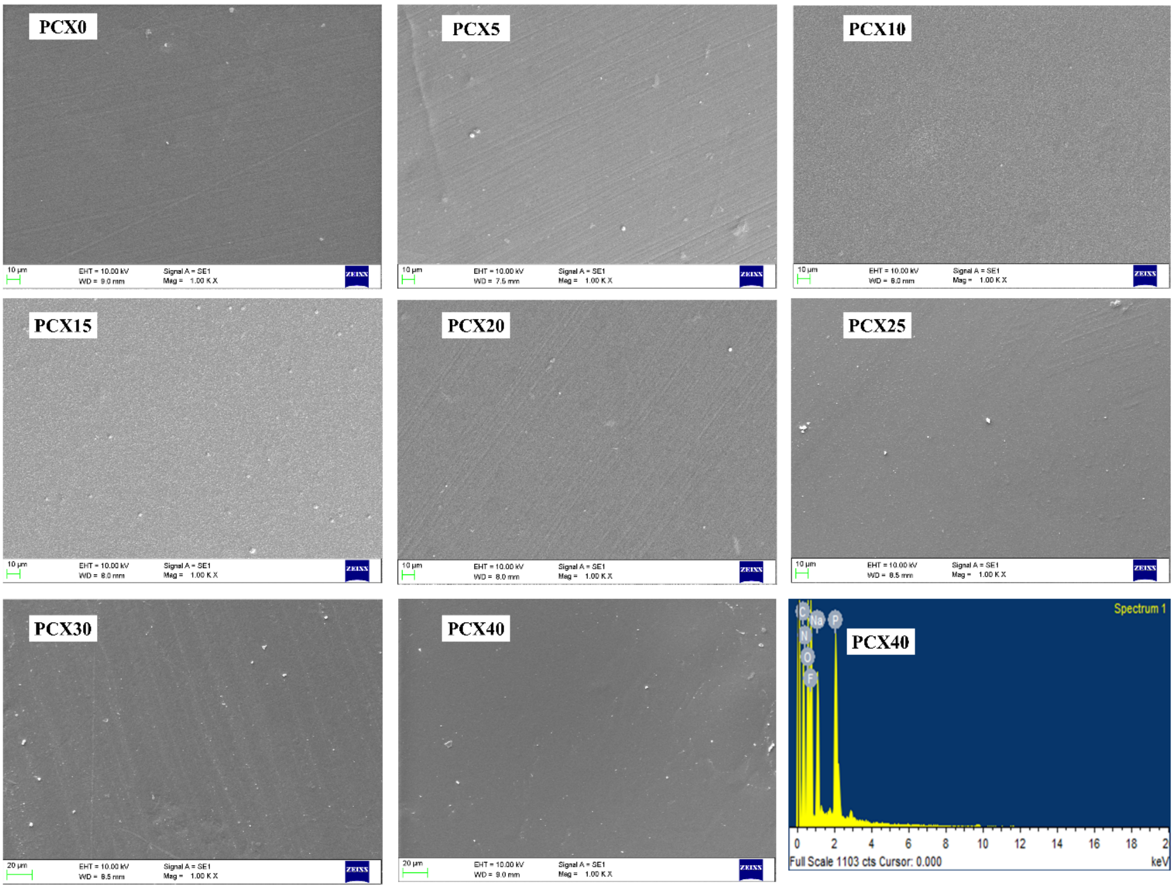

Figure S5: SEM images of selected PCX SPEs and EDAX spectra of PCX40 (at bottom right)

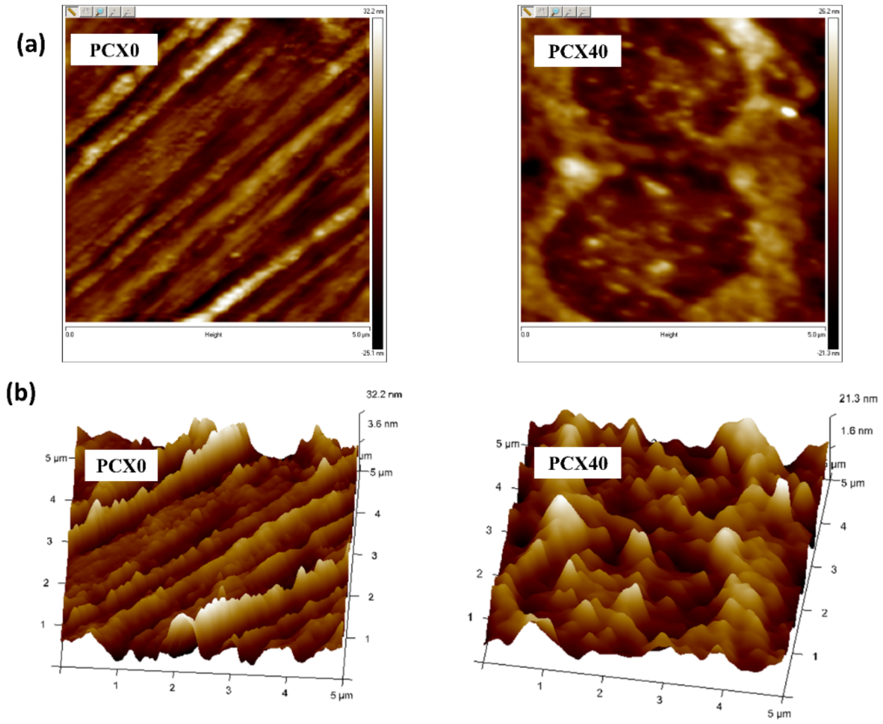

Figure S6: AFM 2D and 3D images of PCX0 and PCX40 SPEs

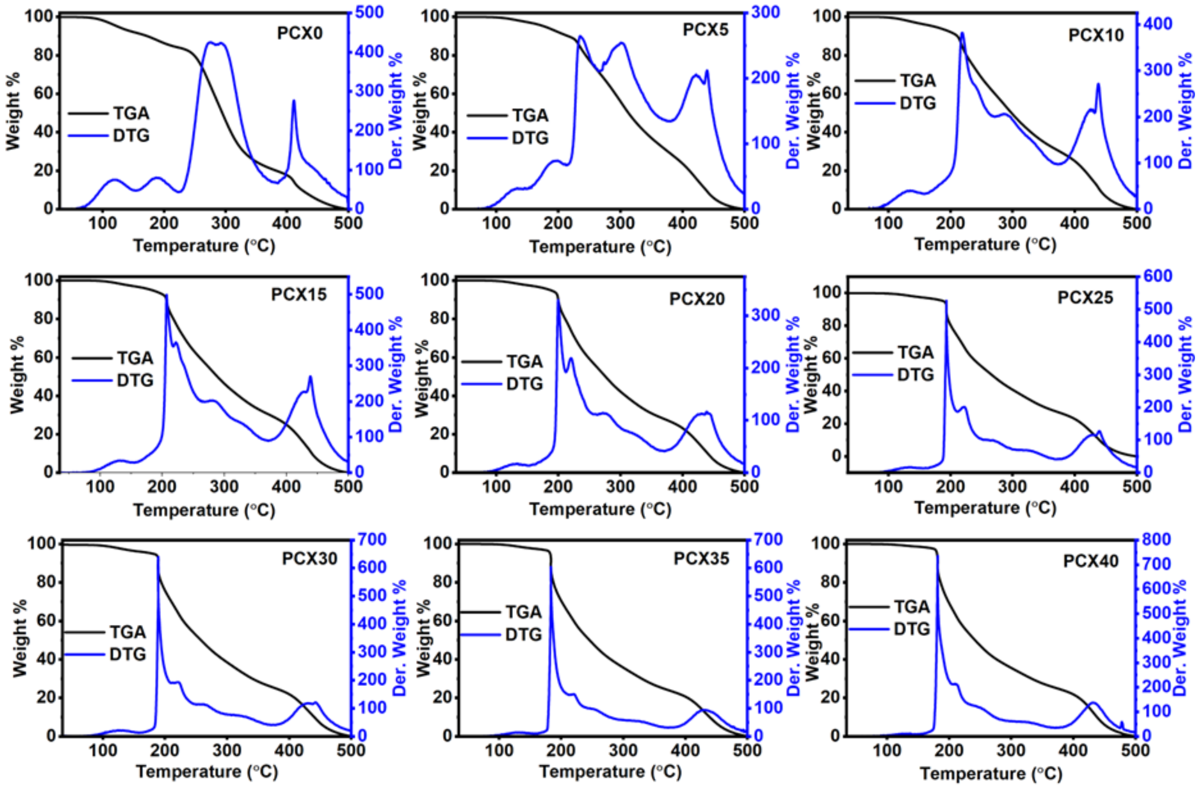

Figure S7: TGA and DTG plots of PVA/CS-$NaPF_6$ SPEs

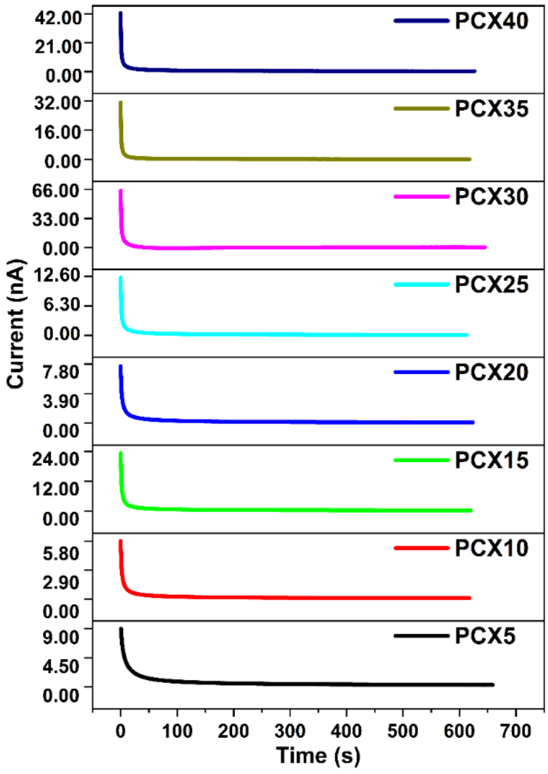

Figure S8: Chronoamperometry (CA) plots PVA/CS-$NaPF_6$ SPEs

Table S1: Values of t_{ion} and σ_{ion} for prepared SPEs obtained from TNM measurements.

Sample	t_{ion}	σ_i(S cm^{-1})
PCX5	0.971	1.672×10^{-7}
PCX10	0.984	3.767×10^{-7}
PCX15	0.985	7.757×10^{-7}
PCX20	0.986	1.819×10^{-6}
PCX25	0.992	6.110×10^{-6}
PCX30	0.997	1.277×10^{-5}
PCX35	0.996	5.475×10^{-5}
PCX40	0.992	6.881×10^{-5}

Table S2: Mechanical properties of PVA/CS-$NaPF_6$ SPEs

Sample	Tensile strength (MPa)	Elongation at break (%)	Young's modulus (MPa)
PCX0	55 ± 2.6	8.1 ± 1.1	2.1E3 ± 7.0 E2
PCX5	32 ± 4.5	27 ± 3.6	5.1E3 ± 1.1E2
PCX10	27 ± 1.8	27 ± 2.7	3.3E2 ± 3.7
PCX15	24 ± 11	27 ± 11	2.0E2 ± 25
PCX20	21 ± 6.2	30 ± 4.6	1.5E2 ± 39
PCX25	13 ± 5.3	24 ± 8.1	70 ± 30
PCX30	13 ± 5.0	37 ± 14	54 ± 9.9
PCX35	6.1 ± 0.89	23 ± 1.4	41 ± 3.9
PCX40	7.3 ± 0.65	31 ± 2.7	29 ± 1.3
